# Production of Epoxyketone Peptide-Based Proteasome Inhibitors by *Streptomyces* sp. BRA-346: Regulation and Biosynthesis

**DOI:** 10.3389/fmicb.2022.786008

**Published:** 2022-03-24

**Authors:** Bruna Domingues Vieira, Henrique Niero, Rafael de Felício, Luiz Fernando Giolo Alves, Cristina Freitas Bazzano, Renata Sigrist, Luciana Costa Furtado, Gabriela Felix Persinoti, Leticia Veras Costa-Lotufo, Daniela Barretto Barbosa Trivella

**Affiliations:** ^1^Brazilian Biosciences National Laboratory (LNBio), Brazilian Center for Research in Energy and Materials (CNPEM), Campinas, Brazil; ^2^Faculty of Pharmaceutical Sciences (FCF), University of Campinas (UNICAMP), Campinas, Brazil; ^3^Institute of Computing (IC), University of Campinas (UNICAMP), Campinas, Brazil; ^4^Department of Pharmacology, Institute of Biomedical Sciences, University of São Paulo, São Paulo, Brazil; ^5^Brazilian Biorenewables National Laboratory (LNBR), Brazilian Center for Research in Energy and Materials (CNPEM), Campinas, Brazil

**Keywords:** *Streptomyces* sp. BRA-346, epoxyketone peptides, proteasome inhibitors, genome mining, biosynthesis of natural products, transformation-associated recombination cloning, mass spectrometry, molecular networking

## Abstract

*Streptomyces* sp. BRA-346 is an Actinobacteria isolated from the Brazilian endemic tunicate *Euherdmania* sp. We have reported that this strain produces epoxyketone peptides, as dihydroeponemycin (DHE) and structurally related analogs. This cocktail of epoxyketone peptides inhibits the proteasome chymotrypsin-like activity and shows high cytotoxicity to glioma cells. However, low yields and poor reproducibility of epoxyketone peptides production by BRA-346 under laboratory cultivation have limited the isolation of epoxyketone peptides for additional studies. Here, we evaluated several cultivation methods using different culture media and chemical elicitors to increase the repertoire of peptide epoxyketone production by this bacterium. Furthermore, BRA-346 genome was sequenced, revealing its broad genetic potential, which is mostly hidden under laboratory conditions. By using specific growth conditions, we were able to evidence different classes of secondary metabolites produced by BRA-346. In addition, by combining genome mining with untargeted metabolomics, we could link the metabolites produced by BRA-346 to its genetic capacity and potential regulators. A single biosynthetic gene cluster (BGC) was related to the production of the target epoxyketone peptides by BRA-346. The candidate BGC displays conserved biosynthetic enzymes with the reported eponemycin (EPN) and TMC-86A (TMC) BGCs. The core of the putative epoxyketone peptide BGC (ORFs A-L), in which ORF A is a LuxR-like transcription factor, was cloned into a heterologous host. The recombinant organism was capable to produce TMC and EPN natural products, along with the biosynthetic intermediates DH-TMC and DHE, and additional congeners. A phylogenetic analysis of the *epn/tmc* BGC revealed related BGCs in public databases. Most of them carry a proteasome beta-subunit, however, lacking an assigned specialized metabolite. The retrieved BGCs also display a diversity of regulatory genes and TTA codons, indicating tight regulation of this BGC at the transcription and translational levels. These results demonstrate the plasticity of the *epn/tmc* BGC of BRA-346 in producing epoxyketone peptides and the feasibility of their production in a heterologous host. This work also highlights the capacity of BRA-346 to tightly regulate its secondary metabolism and shed light on how to awake silent gene clusters of *Streptomyces* sp. BRA-346 to allow the production of pharmacologically important biosynthetic products.

## Introduction

**Figure 1:**
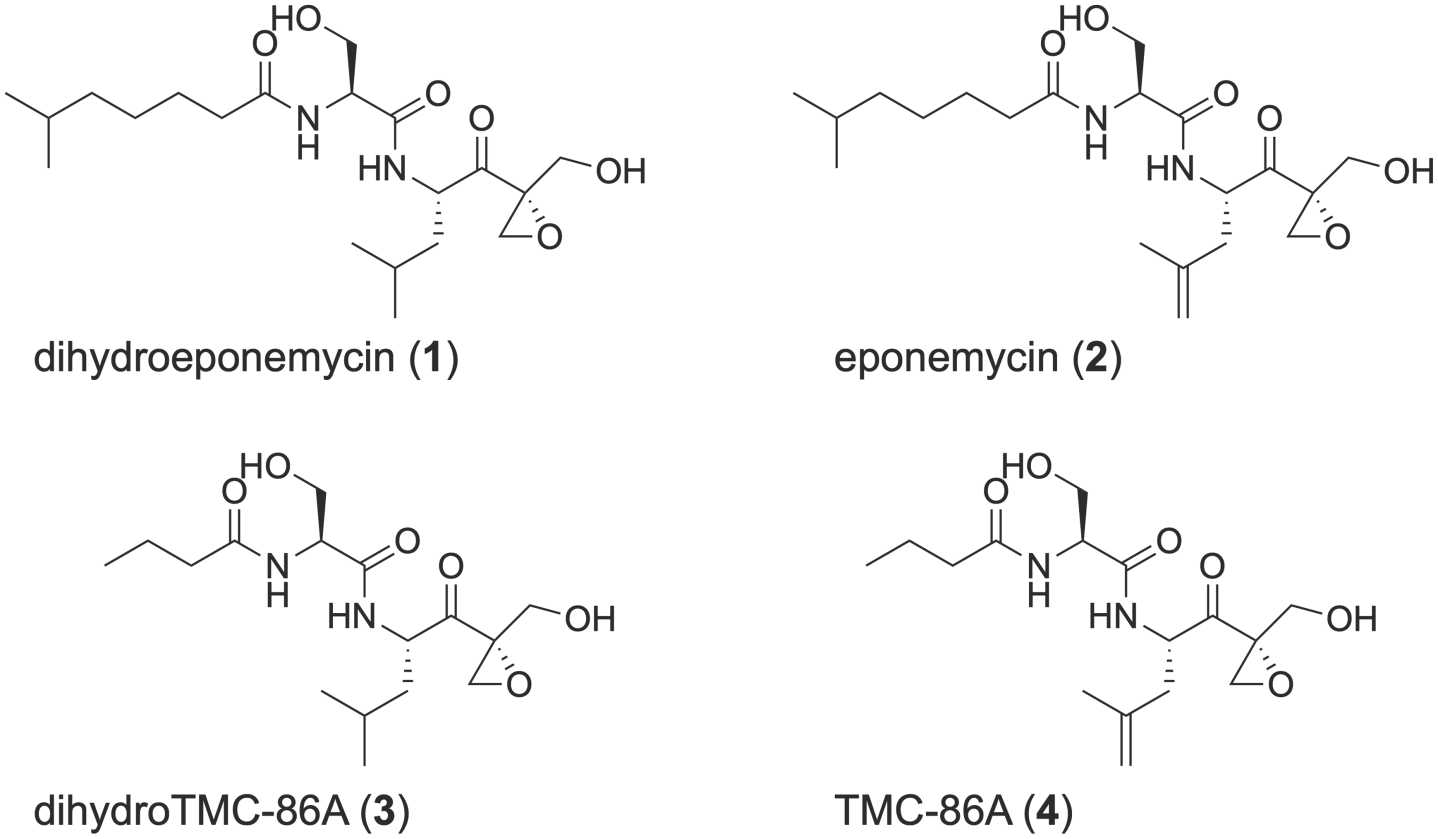
Chemical structures of the epoxyketone peptides discussed in this work.

Marine *Streptomyces* sp. BRA-346, isolated from the Brazilian endemic tunicate *Euherdmania* sp., produces epoxyketone peptides, such as dihydroeponemycin (DHE, 1), eponemycin (EPN, **2**), dihydro TMC-86A (DH-TMC, **3**), and TMC-86A (TMC, **4**; Figure 1). Both DHE and an epoxyketone-containing enriched fraction were evaluated by Furtado et al. (2021) in glioma cell lines, displaying high cytotoxicity to HOG and T98G cells (GI_50_ of 1.6 and 1.7 ng/ml for DHE, and 17.6 and 28.2 ng/ml for the BRA-346 fraction, respectively). The enriched fraction inhibited the proteasome ChTL activity with IC_50_ of 45 ng/ml (Furtado et al., 2021). Additional studies showed that the epoxyketone-containing fraction (at GI_50_ levels) led to the accumulation of ubiquitinated proteins and upregulation of genes related to ER-stress response, correlating to the observed proteasome inhibition. DHE alone induced similar effects in treated cells, but at concentrations 25 times its GI_50_, suggesting that the other epoxyketone compounds contained in *Streptomyces* sp. BRA-346 samples might boost proteasome inhibition and further cellular effects in glioma cells. These findings reinforced the potential of these marine bacteria in producing a cocktail of structurally related compounds that affect the viability of glioma cells through proteasome inhibition.

The proteasome is an enzymatic protein complex of ~750 kDa, which is responsible for non-lysosomal cell proteolysis. Natural and synthetic molecules have been shown to selectively inhibit the proteasome, especially its chymotrypsin-like catalytic activity. These proteasome inhibitors selectively kill cancer cells by promoting protein stress and inducing apoptosis in highly replicating and protein synthesis active cells (reviewed by Kisselev et al., 2012). Proteasome inhibitors were first approved for treating multiple myeloma (MM) and still represent one of the best alternatives for treating MM (Moreau et al., 2012; Hungria et al., 2019; Ito, 2020). More recently, proteasome inhibitors are also being repositioned to solid tumors (Roeten et al., 2018) as glioblastoma (Huang et al., 2017; Roth et al., 2020), and breast cancer (Jones et al., 2010; Wang et al., 2016a; Weyburne et al., 2017). Peptide epoxyketone proteasome inhibitors such as the natural eponemycin (Sugawara et al., 1990; Meng et al., 1999), epoxomicin (Hanada et al., 1992; Kyung et al., 1999; Groll et al., 2000), carmaphycin (Pereira et al., 2012; Yang et al., 2013; Trivella et al., 2014), and the synthetic approved drug carfilzomib (Fostier et al., 2012) inhibit the proteasome with high potency and selectivity. They are irreversible inhibitors of the three proteasome proteolytic activities. DHE (**1**), EPN (**2**), and further analogs were found in BRA-346 cultures, from which the crude extract and an enriched fraction showed potent proteasome and cancer cell growth inhibition (IC_50_ < 20 ng/ml; Furtado et al., 2021). The diversity of eponemycin analogs found in BRA-346 cultures stimulated further investigation on their biosynthesis and regulation.

Compound production by bacteria can be tightly regulated. It is estimated that less than 10% of the bacterial genome capacity is expressed under laboratory conditions (Rutledge and Challis, 2015; Liu et al., 2021). This is due to BGC expression regulation by specific BGC regulators (Fuqua et al., 1994; Santos et al., 2012; Rajput and Kumar, 2017) and also by broader epigenetic modulation (Trivella and de Felicio, 2018; Tomm et al., 2019). The “one strain many compounds” (OSMAC) approach, originally introduced by Bode et al. (2002) and recently reviewed by Pan et al. (2019), has shown that chemical elicitation of bacterial cultures by epigenetic modulators, as HDAC inhibitors (Moore et al., 2012), co-cultivation, culture media, and specialized metabolites, as antibiotics, can induce the production of bacterial metabolites, including the production of rare compounds (Bode et al., 2002; Abdelmohsen et al., 2015; de Felício et al., 2021). In addition, genome mining and BGC cloning have provided alternative approaches for controlling and overproduction of bacterial secondary metabolites under laboratory conditions (Rigali et al., 2018; Mukherji et al., 2020; Liu et al., 2021), for example, by deletion of the negative regulators from the cloned BGC (Yamanaka et al., 2014).

Here, we show that epoxyketone peptides production by BRA-346 is modulated by growth conditions and chemical elicitors and can be overexpressed in a heterologous host. BRA-346 can produce a variety of epoxyketone peptides and other classes of secondary metabolites that are also regulated under laboratory conditions. The production of the target compound DHE (**1**) and structurally related analogues, such as DH-TMC (**3**) and TMC-86A (**4**), could be upregulated by the fungal antibiotic ampicillin, used as a chemical elicitor, and culture media containing high salt concentrations and soluble starch as the carbohydrate source. On the other hand, epoxyketone peptide production by BRA-346 is downregulated when other specialized metabolites are produced by this bacterium. The biosynthetic gene cluster coding for BRA-346 epoxyketone peptides was located by mining the draft genome of BRA-346, also revealing additional BGCs and a complex repertory of regulatory systems coded in the genome of this bacterium. The *epn/tmc* BGC was cloned into the “antibiotic null” *Streptomyces coelicolor* M1146, showing overproduction of epoxyketone peptides compared to the wild-type organism and the plasticity of the *epn/tmc* BGC of BRA-346 in producing a variety of epoxyketone peptides congeners, including **1**, **3** and **4**, and further obtaining eponemycin (**2**). Phylogenetic analyses of the *epn/tmc* BGC give further insights on the preference for **2** or **4** biosynthesis and reveal horizontal gene transfer of this BGC. In addition, a variety of transcription regulators in phylogenetically related BGCs, point this BGC might be tightly regulated at the transcription and translational levels. In particular, in BRA-346, and in close related BGCs, a LuxR transcription factor was found. The BRA-346 LuxR predicted protein shows an N-terminal PAS-regulatory domain, suggesting an additional sensor regulating the *epn/tmc* BGC transcription in *Streptomyces* BRA-346. The data presented here shows the wild type BRA-346 can produce epoxyketone peptides and a myriad of natural products, being the secondary metabolism tightly regulated by complex regulatory systems, which are in turn influenced by culture media, chemical elicitors and potentially redox balance and aeration. Gamma-butyrolactones (GBL) systems and TetR repressors present in other BGCs coded by the BRA-346 genome appear to be regulated in an opposite direction as the *epn/tmc* BGC, competing with this biosynthetic pathway in the wild-type organism. Biosynthetic precursor competition appears as the main factor controlling BRA-346 specialized metabolome expression, although quorum sensing crosstalk cannot be ruled out. In addition to the heterologous expression of BRA-346 *epn/tmc* BGC in “antibiotic null” strains as *S. coelicolor* M1146, eventual artificial control of its LuxR transcription factor are alternatives for further improving biotechnological production of epoxyketone peptides encoded by the *epn/tmc* BGC.

## Materials and Methods

### Strains and Plasmids

Bacterial/yeast strains, and plasmids used in the present study are listed in Supplementary Table S1. The composition of all culture media used in the present study is listed in Supplementary Table S2.

### BRA-346 Cultures

For standard experiments *Streptomyces* sp. BRA-346 was grown in 25 ml of A1 liquid medium with 1 ml of 20% (v/v) mycelial stock at 28°C for 48 h with shaking. Then, 1 ml of the seed culture was inoculated into 500 ml flasks (equipped with helical springs) with 100 ml of A1 liquid medium. Cultures were grown for 7 days at 28°C and 220 rpm. To test different media, BRA-346 was grown in A1, TSB, TSBY, or ISP2 media under the same conditions.

### Elicitation Experiments

Testing of chemical elicitors was performed as described previously (de Felício et al., 2021). Briefly, BRA-346 was inoculated in 10 ml of A1 medium at 28°C, 200 rpm for 48 h. One-hundred microliter of culture was plated onto SFM agar media and incubated at 30°C for 72 h. The chemical elicitors were added to the solid media plates using an automated pipette to dispense 5 μl drops of each chemical elicitor onto the bacteria layer. The chemical elicitors used were: ampicillin 100 or 10 μg/ml, chloramphenicol 300 or 30 μg/ml (prepared in ethanol), kanamycin 100 or 10 μg/ml, streptomycin sulfate 50 or 5 μg/ml, sodium butyrate 50 or 5 μM, procaine 100 or 10 μM, DMSO 100 or 2% or EDTA 10 or 1 mM. Water and ethanol were used as controls. Plates were incubated at 30°C for 72 h. Alterations on colony morphology were visually inspected and recorded, following the described in de Felício et al. (2021), and used to select the chemical elicitors for liquid culture. The liquid cultures for the elicitation tests were performed as described above, with the addition of the chemical elicitors 72 h after BRA-346 inoculum.

### DNA Sequencing, Assembly, Annotation, and Genome Mining

The BRA-346 genomic DNA was extracted using the Wizard® Genomic DNA Purification Kit (Promega Corporation, Fitchburg, United States). Before sequencing, the quality of the extraction was verified by agarose gel electrophoresis. The genomic DNA was sequenced by the Macrogen laboratory (South Korea, Seoul), using the HiSeq System platform (Illumina Inc., San Diego, United States). Paired-end reads were quality checked using FastQC and processed with Trimmomatic (Bolger et al., 2014) to remove low quality reads and adapters sequences.^1^ QC reads were *de novo* assembled using the A5-miseq pipeline (Coil et al., 2015). Genome quality and completeness were evaluated using both Quast and CheckM (Gurevich et al., 2013; Parks et al., 2015). Gene prediction and annotation were performed with Prokka (Seemann, 2014) and BGCs were further predicted using the antiSMASH web server version 6.0.1 (Blin et al., 2021). The draft genome of *Streptomyces* sp. BRA-346 is available at GenBank under the BioProject accession number PRJNA765318.

### Genetic Manipulation and TAR Cloning

The *epn/tmc* BGC was cloned by PCR-based transformation-associated recombination (TAR) method, as described previously (Zhang et al., 2019; Sigrist et al., 2020), with minor modifications as detailed in the Supplementary Material.

### *Streptomyces coelicolor* M1146 and M1146-*epn/tmc* Cultures

*Streptomyces coelicolor* M1146 (host) and M1146-*epn/tmc* (heterologous organism) were cultivated in A1 medium for 72 h at 28°C and 220 rpm (100 ml in 500 ml flasks equipped with helical springs). M1146 and M1146-*epn/tmc* cultures were supplemented with 25 μg/μl nalidixic acid, and M1146-*epn/tmc* was also supplemented with 50 μg/ml kanamycin.

### Extraction and Fractionation

The cultures of *Streptomyces* sp. BRA-346 (100 ml), *S. coelicolor* M1146 (100 ml), and M1146-*epn/tmc* (100 ml), along with the culture medium A1 (control), were extracted by liquid–liquid partition with ethyl acetate. The biomass was filtered with glass filters (22 μm) after adding the solvent and before phase separation. The extraction was repeated twice by adding 40 ml of ethyl acetate to 100 ml of BRA-346 cultures. The organic phase was collected and dried under reduced pressure at 35°C. The crude extracts were submitted to solid-phase separation using C8 cartridge (Discovery® DSC-8 SPE Tube, Supelco) using H_2_O:MeOH gradient, yielding three fractions: 5% (F5), 50% (F50), and 100% (F100) of methanol. Crude extract and fraction samples were resuspended in DMSO to a final concentration of 10 mg/ml. All samples were analyzed by UPLC–MS/MS.

### LC–MS/MS Data Collection

LC-MS/MS data collection was performed as described previously (de Felício et al., 2021). For UPLC–MS/MS chemical profile, 2 μl aliquots of the samples (extracts and fractions) were injected on a BEH C18 reversed-phase column (1.7 μm, 2.1 × 100 mm) attached to a compatible pre-column, using an Acquity H-Class UPLC Waters (Waters, Milford, MA, United States) system coupled to a Bruker Impact II UHR-ESI-QqTOF mass spectrometer (Bruker Daltonics, Billerica, MA, United States). A solvent system of water (A), acetonitrile (B) 2% formic acid (C) methanol (D), was used to compose the following analytical method: 0.5 ml/min flow rate; column temperature 40°C; 0–1 min, 5% B; 1–6 min, 5% B to 35% B (curve 6); 6–10 min, 35% B to 95% B (curve 1); 10–12 min, 95% B; 12–15 min, 95% D (curve 1); 15–18 min for column equilibration on initial phase. C was kept constant, at 5% (final concentration of 0.1%). The mass spectrometer worked in positive ion mode scanning mass in the 30–2,000 Da range, with acquisition rate of 8 Hz. End plate offset = 500 Volts (V); V_cap_ 4,500 V; nebulizer 4.0 bar; drying gas (N_2_) flow 10 L/min; drying gas temperature 200°C, followed by an MS/MS scan for the most intense ions in a cycle time of 1 s, absolute threshold (per 1,000 sum.) of 1,500 cts. As MS^2^ rules, mass ratio (*m/z*) below 200 Da were excluded, and the “active exclusion” function was enabled. Each run was automatically calibrated using HCOONa (10 mM). Calibrated spectra were converted to mzXML files through Data Analysis 4.3 and included bio tools CompassXport (Bruker Daltonics, version 4.0.0.8).

### NP^3^ MS Workflow

Data processing and analysis were done with an in-house collection of scripts called NP^3^ Mass Spectrometry (MS) Workflow, as previously described (de Felício et al., 2021) with some parameter modifications to adjust sample properties. The beta version of the NP^3^ MS workflow is freely available in our repository (accessed on 13 Aug 2021) with a detailed manual and a command line interface.^2^ The Ionization Variant Annotation Molecular Networking (IVAMN) was used as the base to select and exclude nodes present in chromatographic blanks (BLANKS) samples and their first immediate neighbors. Then, the remained nodes marked as “protonated representative” (column “protonated_representative” equals 1 at the nodes count table) were selected and filtered at the Spectra Similarity Molecular Network (SSMN). The raw SSMN was created with a minimum cosine similarity score for an edge connection of 0.6 and filtered with a maximum component size of 200 and a maximum number of connections for one single node of 15. IVAMN and SSMN construction at Cytoscape (Shannon et al., 2003) were done with an in-house python script that automates some tasks using the py2cytoscape library (accessed on 13 Aug 2021).^3^ After these automated steps, some other undesirable nodes like the ones marked as culture media (BED) and the single nodes were manually removed leaving a clean network to process and analyze. MS/MS chemical structure annotation was performed using spectra matches to the GNPS (Wang et al., 2016b) and the UNPD-ISDB (Allard et al., 2016) databases. Annotations were manually revised and the related MS/MS clusters were grouped and named by the chemical groups found in the SSMN.

### BRA-346 *epn/tmc* BGC Phylogenetic Analysis

Sequences were collected from antiSMASH *ClusterBlast* and *KnownClusterBlast* (Blin et al., 2021), from the antiSMASH-database (*ClusterBlast* Hit and *KnownClusterBlast* Hit for experimentally validated epoxyketone peptides BGCs; Blin et al., 2017), from the BiG-FAM database (querying the 29,955 Biosynthetic Gene Families, previously calculated using BiG-SLICE from a collection of more than 1.2 million Biosynthetic Gene Cluster; Kautsar et al., 2021a,b), and from MultiGeneBlast (Medema et al., 2013) run on the bacteria GenBank database from 2013 and a custom *Streptomyces* database from GenBank (2,526 NCBI GenBank Assemblies), using BRA-346 *epn/tmc*, *Streptomyces hygroscopicus* eponemycin and *S. chromofuscus* TMC-86A BGCs as probes. The best hit sequences, indicated by their closest relationship with BRA-346 *epn/tmc* BGC (low distance and high cumulative BLAST hit score), all carrying the acyl-CoA dehydrogenase gene, were aligned with BRA-346 *epn/tmc* BGC at nucleotide level with the multiple sequence alignment MAFFT v11 program (Katoh and Standley, 2013) using Geneious Prime (v.2021.2.2). Regions of poor alignment were inspected, and sequence refinement was done with Trimal software (Capella-Gutierrez et al., 2009; Method: Automated1). Construction of maximum likelihood trees from the alignment was conducted using the Molecular Evolutionary Genetics Analysis (MEGA) v11 software (Tamura et al., 2021) after statistical selection of the best-fit model of evolution with jModelTest2 tool (Darriba et al., 2012).

## Results

### Epoxyketone Peptides Production by *Streptomyces* sp. BRA-346 Under Laboratory Conditions

**Figure 2:**
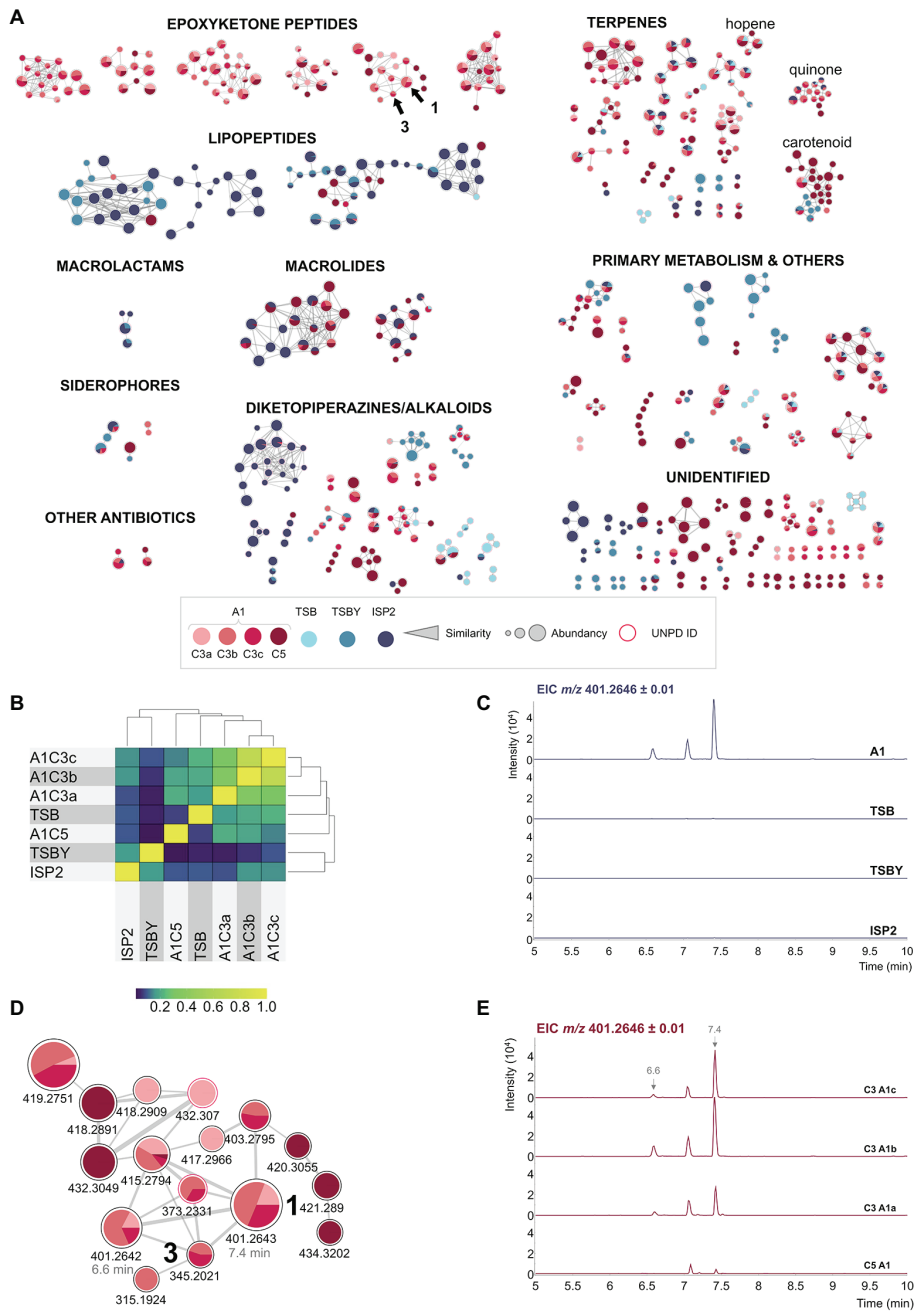
The biosynthesis of BRA-346 secondary metabolites is tightly regulated by the culture media used for bacteria growth. **(A)** Spectra similarity molecular network (SSMN) of the different BRA-346 cultures media A1 (cultures C3a–c and C5, in red tones), TSB (cyan), TSBY (light blue), or ISP2 (dark blue). MS/MS spectra detected in the crude extracts are shown as the network nodes. For clarity, only the MS/MS spectra pointed as the protonated ion ([M + H]^+^) and nodes with at least one connection (self-loops and single nodes were removed) are shown. The MS/MS spectra were annotated by spectra matches with UNPD-ISDB and GNPS databases, visually inspected and compared to literature data. Chemical classes were annotated, and the MS/MS clusters grouped accordantly. The annotated chemical classes could also be linked to the *Streptomyces* sp. BRA-346 draft genome. DHE **(1)** and DH-TMC **(3)** are pointed by arrows. The MS/MS annotations for **1** and **3** were based on the described by Furtado et al. (2021) and Zabala et al. (2016), respectively. The parameter used for node size was intensity value ([minimum value; 100,000]: linear interpolation, [100,000; maximum value]: constant size). **(B)** Heat map constructed from all nodes detected in the SSMN shown in (A), considering the seven BRA-346 cultures analysed. The MS1 peak area was normalized by the maximum area of a given *m/z* and used for a cosine similarity analysis of the detection of each node across the different cultures. **(C)** Extracted ion chromatogram (EIC) of *m/z* 401.26 ± 0.01 in BRA-346 cultures with the different media used. The region 5–10 min (“x” axis) was selected for clarity. **(D)** Detail of the epoxyketone peptide MS/MS cluster extracted from the SSMN shown in **(A)**. **(E)** EIC of *m/z* 401.26 ± 0.01 in the four BRA-346 cultures in the A1 medium.

BRA-346 is a slow growing Actinobacteria, which achieves the stationary phase after at least 3 days of laboratory growth in highly aerated liquid cultures. Secondary metabolite production is expected by day 7, with epoxyketone peptides production by BRA-346 being culture and medium-dependent (Figure 2).

To exemplify this statement, four replicates of BRA-346 cultures in the A1 medium, the standard growth medium for BRA-346 (Furtado et al., 2021), were carried out (A1 C3a-c and A1 C5), in addition to the use of three different culture media (ISP2, TSBY, and TSB; Supplementary Table S2), in a total of seven culture conditions evaluated in this data set. The metabolome was assessed by LC–MS/MS analyses, being further evaluated by the construction and inspection of a spectra similarity molecular network (SSMN) of the representative [M + H]^+^ ions (de Felício et al., 2021; Figure 2A).

Notably, BRA-346 metabolome is shifted towards different classes of secondary metabolites, depending on the culture media used (Figures 2A,B). Specifically, the production of the target epoxyketone peptides was upregulated in the cultures using the A1 medium and downregulated when the other culture media were applied (Figures 2A,C). The downregulation of epoxyketone peptide production is concomitant with the overexpression of lipopeptides and macrolactams in the ISP2 and TSBY culture media (Figure 2A). In addition, the A1 C5 culture also presented a reduced diversity of epoxyketone peptides (Figures 2D,E), which was in turn aligned with an increase detection of lipopeptides, macrolides, and carotenoids (Figure 2A).

The epoxyketone peptide cluster containing **1** and **3** is highlighted in Figure 2D, spotting DHE (**1**, *m/z* 401.26) and DH-TMC (**3**, *m/z* 345.20). Two ions of *m/z* 401.26, which were clustered together in the SSMN, presented different retention times in the reverse-phase liquid chromatography (6.6 min and 7.4 min; Figure 2E). Their fragmentation spectra (Supplementary Figure S5) are similar (cosine = 0.88), suggesting that these ions represent stereoisomers. It is also important to comment that the bioactive epoxyketone peptide eponemycin (**2**, *m/z* 399.25), which was previously identified in BRA-346 cultures in low yields (Furtado et al., 2021), was not detected in the crude extracts of BRA-346 cultures presented here (Supplementary Figures S6, S7). In addition, TMC-86A (**4**, *m/z* 343.19), was identified in trace amounts (Supplementary Figure S8). Knowing that **1** and **3** are biosynthetic intermediates of **2** (Schorn et al., 2014) and **4** (Zabala et al., 2016), respectively, it is possible that the last step of **2** and **4** biosynthesis in wild type BRA-346 is not being completed under the growth conditions used.

### Chemical Elicitors Induced Epoxyketone Production by BRA-346

**Figure 3:**
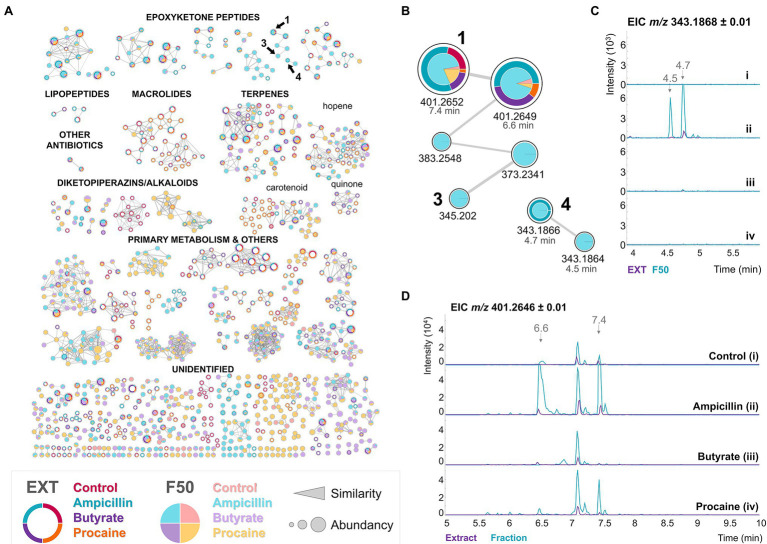
Chemical elicitors, especially ampicillin, induce the production of epoxyketone peptides by BRA-346. **(A)** SSMN of BRA-346 cultures using the A1 medium and different chemical elicitors [no elicitor, as a control, (i); ampicillin 100 μg/ml (ii); sodium butyrate 50 μM (iii); procaine 100 μM (iv)]. Only the [M + H]^+^ spectra and nodes with at least one connection are shown for clarity. In each node, the external ring chart represents the crude extracts, and the internal pie chart represents the F50 (50% methanol) fractions. **(B)** Detail of the epoxyketone MS/MS clusters containing compounds **1**, **3** and **4**. **(C)** and **(D)** represent the EIC of *m/z* 343.19 ± 0.01 (TMC, **4**) and *m/z* 401.26 ± 0.01 (DHE, **1**), respectively. Chromatograms of the crude extract and F50 fractions samples are represented in purple and cyan, respectively.

In a further attempt to elicit secondary metabolite production by BRA-346, especially epoxyketone peptides, we used chemical elicitors in A1 cultures. This was done following precedents in the literature that some chemicals, as sodium butyrate (reviewed by Tomm et al., 2019), can act as epigenetic modulators, in addition to mimetics of the presence of competing organisms, as antibiotics produced by other micro-organisms (Abdelmohsen et al., 2015; de Felício et al., 2021). Following the approach described by de Felício et al. (2021) we tested several chemical elicitors in solid media, and selected ampicillin (100 μg/ml), sodium butyrate (50 μM), and procaine (100 μM) for eliciting BRA-346 secondary metabolites in A1 liquid medium. The metabolites were extracted, and the crude extracts were analysed by LC–MS/MS and SSMN (Supplementary Figure S9). The experiments (C5) were carried out in parallel, in the same incubator, and following the same procedures. In addition, the crude extracts were purified using C-8 cartridges and the water/methanol fractions (F50) were further analyzed (Figure 3).

It is worth noticing that **1** was produced in higher amounts in the ampicillin elicited cultures (Figure 3D), and TMC-86A (**4**), which had not been previously detected or sporadically detected in trace amounts (Figure 3C), was detected in the crude extracts and enriched in the F50 fraction of the ampicillin growth condition (Figure 3B). These results indicated that BRA-346 could produce **4**, one of the expected final products of epoxyketone biosynthesis in *Streptomyces* bacteria. However, its production might be tightly regulated in BRA-346 and can be upregulated by an antibiotic chemical elicitor.

Analysing the groups of metabolites modulated by the chemical elicitors (Supplementary Figure S9), ampicillin increased epoxyketone and downregulated macrolides, terpenes, and diketopiperazines/alkaloids. Procaine, on the other hand, exerted an opposite effect, downregulating epoxyketone peptides and upregulating the three other major groups (macrolides, terpenes, and diketopiperazines/alkaloids). Macrolactams were not observed and just a small group of lipopeptides was observed.

### Draft Genome Sequence of *Streptomyces* BRA-346

Our efforts to understand epoxyketone biosynthesis and regulation by *Streptomyces* BRA-346 were further assisted by genome sequencing and mining, going beyond this class of secondary metabolites. BRA-346 draft genome contains 10 Mb and was presented on 180 contigs with 8,706 predicted coding genes.

BRA-346 genome mining and annotation using antiSMASH (Blin et al., 2021) show the presence of 66 putative biosynthetic gene clusters (BGCs). Although this is a draft genome, and some clusters are fragmented or duplicated, it was possible to find 35 complete BGCs encoding for ribosomally synthesized and post-translationally modified peptides (RiPPs), epoxyketone peptides, terpenes, ectoines, siderophores, non-ribosomal peptides (NRP) or polyketides (PK; Supplementary Table S6). In addition, 31 truncated BGCs encoding for PK or NRP were also annotated. This reflects a broad biosynthetic capacity of BRA-346, which is mostly hidden under laboratory conditions.

By applying the OSMAC (one strain many compounds) approach, using specific growth conditions (e.g., culture media—Figure 2A; Supplementary Table S4; and chemical elicitors—Figure 3; Supplementary Figure S9), we were able to reveal the different classes of secondary metabolites produced by this bacterium. Some of the BGCs annotated in BRA-346 draft genome could be linked to the metabolites presented in the SSMN (Figure 2A), providing additional insights into BRA-346 regulation and metabolic shifts.

In the regulatory perspective, the draft genome of *Streptomyces* BRA-346 shows a myriad of transcription regulators, which may be involved in the regulation of the specialized metabolism of BRA-346 under laboratory conditions. We highlight 70 LuxR-type transcription regulators, 118 TetR-type regulators, 2 gamma-butyrolactone (GBL) BGCs (region 35.1, candidate BGC = 11 kb; and region 2.1, candidate BGC = 11 kb, Supplementary Table S6; Supplementary Figure S10), which might be of particular importance for understanding BRA-346 regulation and, potentially quorum sensing.

For lipopeptides, which were evidenced in BRA-346 cultures using TSBY/ISP2 media (Figure 2A), a truncated NRPS BGC was found in contig 32 harboring the assembly line characteristic of lipopeptides, thus linking the metabolites detected by LC–MS/MS to BRA-346 genome. This BGC (region 32.1, candidate BGC 35 kb; Supplementary Table S6) contains a N-terminal acyl ligase domain, which is associated with lipopeptide biosynthesis (Kersten et al., 2011), in addition to NRPS modules. It further shares sequence similarity with *Streptomyces* RTd22 (Chagas et al., 2016) and ATCC53653 BGCs, the latter reported previously as a producer of stendomycin lipopeptides—Supplementary Figures S11, S12; Supplementary Table S5 (Kersten et al., 2011; Villadsen et al., 2018). A complex regulatory system is found within this BGC, which harbours TetR, LysR, sensor histidine kinase, SARP, and LuxR regulators.

Macrolactams, exemplified by heronamide B (with related ORFs present in two truncated regions: region 2.1, candidate BGC = 11 kb and region 63.1, candidate BGC = 27 kb; Supplementary Table S6) could also be linked to the BRA-346 metabolome (Figure 2A). This class of natural products was also only observed in the TSBY/ISP2 cultures. This BGC also presents a complex regulatory system with TetR, LuxR, LacI, AraC, SARP, and an AsfA butyrolactone producing gene.

Diketopiperazines, related to bicyclomycin (region 3.2, candidate BGC 43 kb), and carotenoids, related to isorenieratene (region 34.1, candidate BGC 21 kb), annotated at the SSMN (Figure 2A), were additionally located in BRA-346 draft genome (Supplementary Table S6). Bicyclomycin could be linked to an alkaloid/diketopiperazine SSMN cluster expressed in the ISP2 medium; whereas carotenoids were mostly observed in the TSBY and A1C5 cultures (Figure 2A) and in A1 cultures induced with procaine (Supplementary Figure S9). Curiously, these differentially expressed BGCs are also under the control of TetR (Table 1), a family of transcription factors, generally repressors, regulated by gamma-butyrolactones (GBL; Cuthbertson and Nodwell, 2013).

**Table 1:** Groups of metabolites found to be regulated together in wild type BRA-346 cultures.

Assigned NP class^a^	Regulatory system^b^	Culture media^c^	Chemical elicitor (A1 medium)^d^	Group^e^
Epoxyketone peptide	LuxR, TTA	Induced in the A1 medium	Induced by ampicillin	**G1**
Macrolide; Carotenoid; Other diketopiperazine/alkaloid	MprA/MprB (AraC family, TetR activators); TetR (activator); unidentified	Induced in some cultures of A1 and ISP2 media. Induced in some cultures of A1 and TSBY media. Induced in some cultures of A1 and TSBY media.	Induced by procaine	**G2**
Lipopeptide; Macrolactam; Diketopiperazines related to bicyclomycin	TetR (repressor), LysR, sensor histidine kinase, SARP, TetR (activator), LuxR; TetR (repressor), LuxR (TTA), LacI, AraC, SARP (butyrolactone BGC); TetR (repressor, activator)	Mostly or exclusively observed in TSBY and/or ISP2. TSBY and ISP2 only. Mostly ISP2	Not observed or suppressed in A1 medium, even in the presence of chemical elicitors	**G3**

a The assigned natural product class is given according to LC–MS/MS characteristics and spectra matches with databases.

b The regulatory systems are predicted from BRA-346 draft genome. TetR repressors and activators were suggested according to the position of the HTH domain (C-terminal HTH = activators, N-terminal HTH = repressors; Cuthbertson and Nodwell, 2013).

c Culture media.

d Chemical elicitors (in A1 medium) regulating the assigned natural product classes are also provided.

e Metabolic groups were assigned (G1, G2, or G3) as indicated.

**Figure 4:**
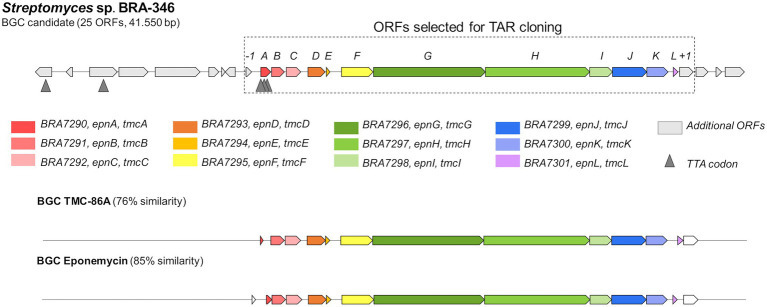
Biosynthetic gene cluster of epoxyketone peptides in BRA-346. The *epn/tmc* BGC in BRA-346 was identified using antiSMASH. The related BGC of eponemycin (*epn*, *Streptomyces hygroscopicus* ATCC53708; Schorn et al., 2014) and TMC-86A (*tmc*, *Streptomyces chromofuscus* ATCC49982; Zabala et al., 2016) already reported are shown for comparison.

For the epoxyketone peptides, mostly observed when BRA-346 was grown in the A1 medium (cultures C3 a–c, Figure 2) or upregulated in the presence of ampicillin (Figure 3; Supplementary Figure S9), a single complete BGC was found (BGC number 42.1, Supplementary Table S6). The BGC, herein named *epn/tmc*, is predicted to contain 25 open reading frames (ORFs). From these, 12 ORFs are related to previously reported eponemycin, *epn* (Schorn et al., 2014) and TMC-86A, *tmc* (Zabala et al., 2016) epoxyketone gene clusters (Figure 4; Supplementary Table S7). This BGC has a simpler regulatory system, compared to the BGCs presented above, with only one LuxR-type transcription factor (*epn/tmcA*) and TTA codons.

### Heterologous Expression of the *epn/tmc* BGC From BRA-346

Although eponemycin (**2**) and TMC-86A (**4**) BGCs in *Streptomyces* bacteria have been already described in the literature, these BGCs were individually reported as producers of eponemycin or TMC-86A natural products. BRA-346 was shown to produce both **1** and **3**, the last step biosynthetic intermediates of **2** and **4**, suggesting multiple epoxyketone BGCs or a multifunctional BGC. In addition, we found the production of these specialized metabolites is tightly regulated in wild type BRA-346 and influenced by its metabolic shifts, limiting their full obtention for additional studies.

**Figure 5:**
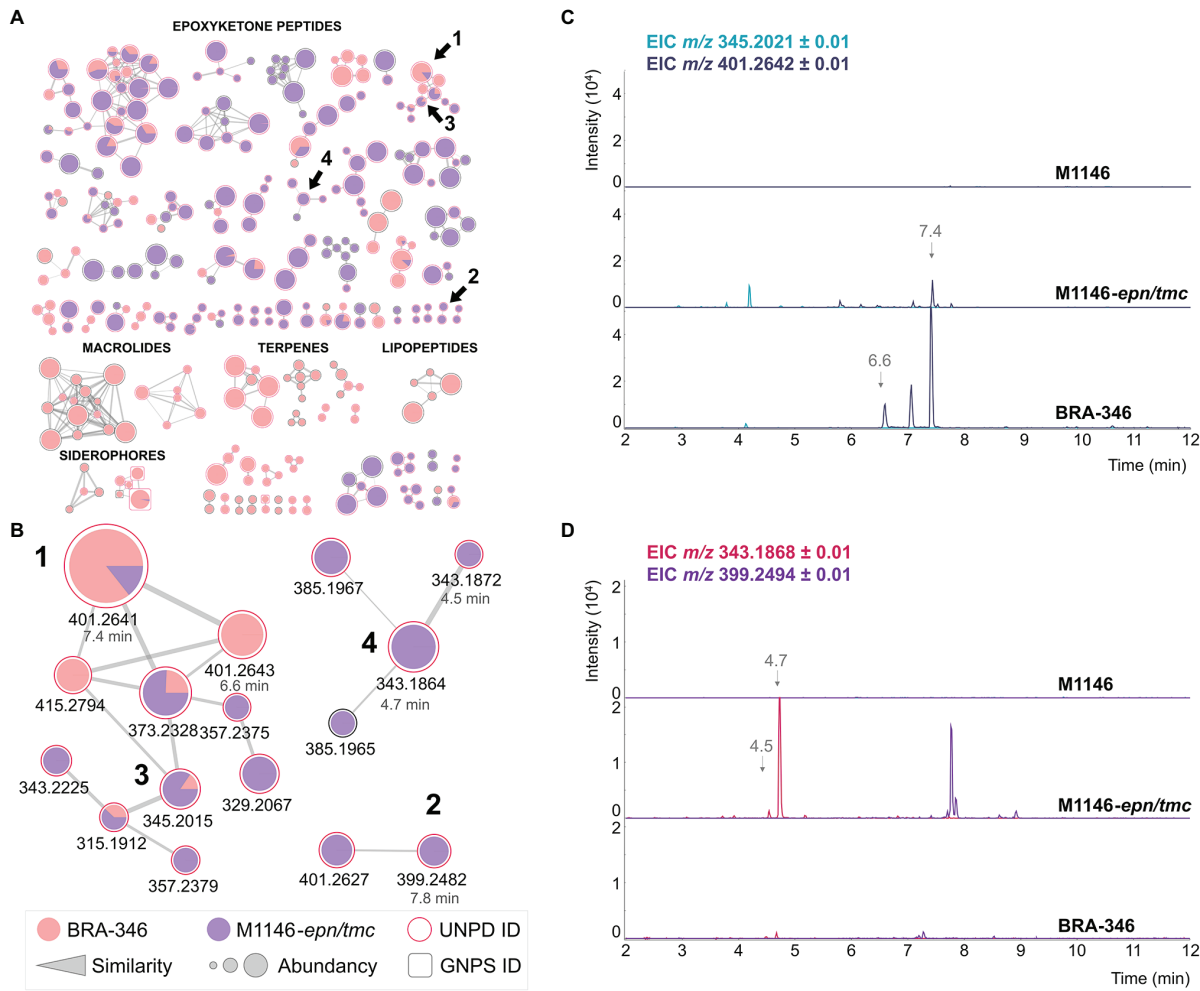
Heterologous expression of the *epn/tmc* BGC of BRA-346. **(A)** SSMN evidencing the production of epoxyketone peptides by the heterologous (purple) and the wild type (pink) organisms. **(B)** Zoom at the SSMN clusters containing compounds **1**–**4**. **(C)** Extracted ion chromatogram of the dihydrointermediates **1** (*m/z* 401.26) and **3** (*m/z* 345.20). **(D)** Extracted ion chromatogram of the final biosynthetic products **2** (*m/z* 399.25) and **4** (*m/z* 343.19).

We then cloned the *epn/tmc* BGC of BRA-346 using the transformation-associated recombination (TAR) approach (Zhang et al., 2019; Sigrist et al., 2020), inserting the target BGC under the control of a strong promoter into a *S. coelicolor* M1146 “antibiotic null” host organism (Gomez-Escribano and Bibb, 2011). The heterologous organism *S. coelicolor* M1446-*epn/tmc* was cultured in A1 medium and its metabolites were analyzed in parallel with the wild-type BRA-346 and the host organism *Streptomyces coelicolor* M1446 (Figure 5A; Supplementary Figure S12). M1146-*epn/tmc* reaches the stationary phase in less than 3 days, with full production of the epoxyketone peptides on day 3, accelerating laboratory cultivation and chemical sample preparation.

The high yields obtained for both final products (**2** and **4**) in the heterologous organism containing the BRA-346 *epn/tmc* BGC (M1146-*epn/tmc*, Figure 5; Supplementary Figure S13) are worth noticing, confirming that the *epn/tmc* BGC of BRA-346 is a multifunctional BGC capable of producing both TMC-86A and EPN. It was further possible to detect several [M + H]^+^ MS/MS clusters related to epoxyketone peptides (grouped in Figure 5A), suggesting a collection of epoxyketone peptides that can be formed from BRA-346 *epn/tmc* BGC expression.

In comparison to the wild-type BRA-346, the heterologous organism M1146-*epn/tmc* showed higher amounts of the biosynthetic final products **2** and **4** and lower amounts of the dihydro leucine intermediates **1** and **3** (Figure 5; Supplementary Figure S13). Although wild-type BRA-346 was reported to produce **2** (Furtado et al., 2021) it did not produce this compound in our experiments (Figure 3A; Supplementary Figures S6, S7), and produced **4** in low yields (Supplementary Figure S8), even when induced by ampicillin (Figure 3C). Together, these results may point to regulation of the last step of **2** and **4** biosynthesis in wild type BRA-346, which was overcome by heterologous expression of the *epn/tmc* BGC in *S. coelicolor* M1146.

Wild type BRA-346 also presented the production of other antibiotics, what in turn can limit the production of epoxyketone peptides by this highly diverse antibiotic producer (Figure 5A in pink). On the other hand, heterologous expression of the *epn/tmc* BGC in an “antibiotic null” strain resulted in epoxyketone peptide biosynthetic final products overproduction. This suggests that the availability of biosynthetic precursors and/or crosstalk of regulatory mechanisms are controlling epoxyketone peptide production by wild type BRA-346.

### Phylogenetic Analyses of the *epn/tmc* BGC From BRA-346

**Figure 6:**
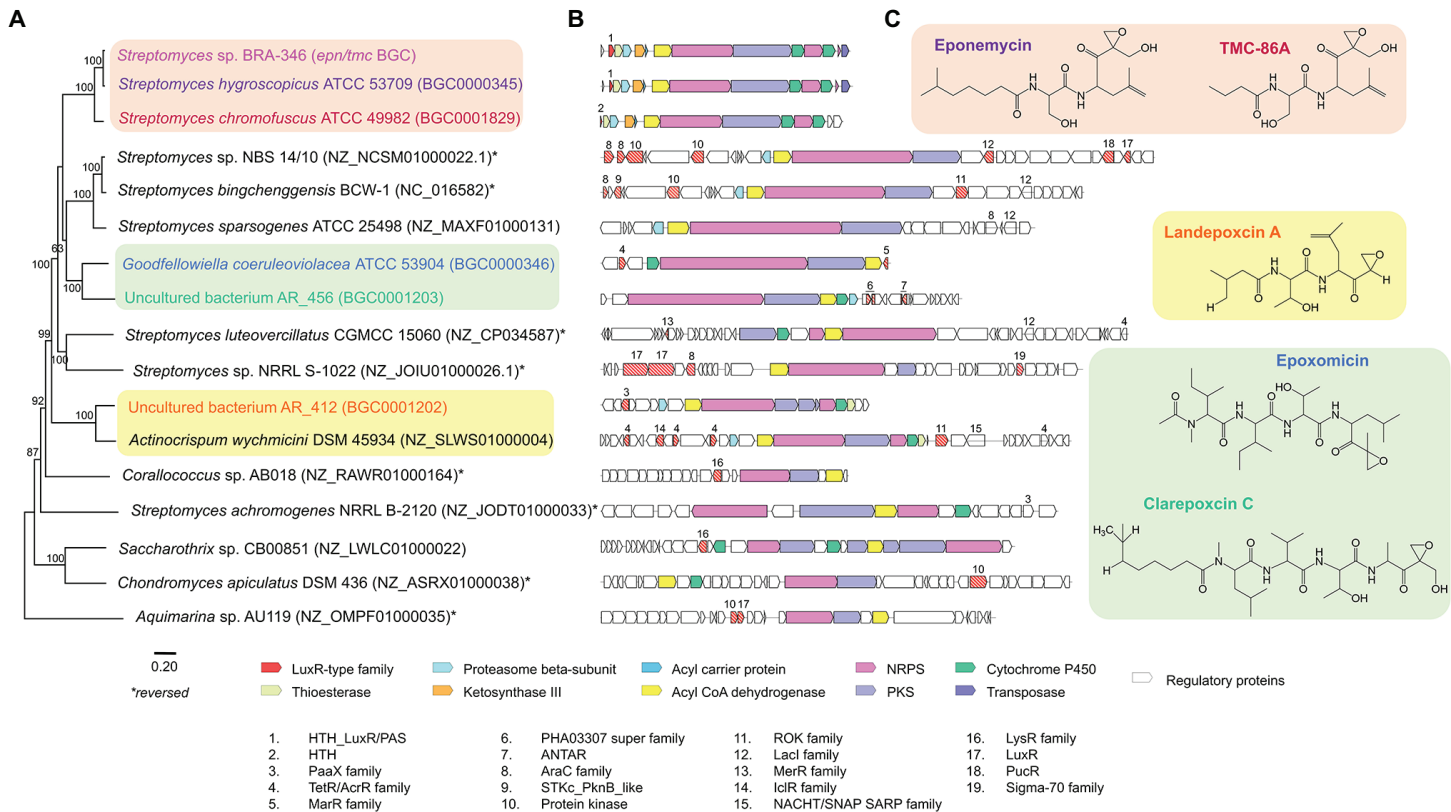
Phylogenetic analysis of BGCs related to BRA-346 *epn/tmc*. **(A)** Maximum likelihood phylogenetic tree based on the alignment of the 16 retrieved BGCs related to BRA-346 *epn/tmc* BGC. **(B)** Schematic representation of the 17 BGCs and **(C)** their related biosynthetic products. BGCs with experimentally validated biosynthetic products are highlighted. ORFs predicted as regulatory proteins are numbered and listed.

Aiming to gain further insights on the occurrence and phylogenetic relationship of the BRA-346 *epn/tmc* BGC we additionally searched for related BGCs in public databases and constructed a phylogenetic tree for this BGC (Figure 6). *Aquimarina* sp. putative BGC was assigned as the outgroup of the tree, since it was the most distant BGC to BRA346 *epn/tmc*, while sharing sequence similarities. This analysis showed that the BRA-346 *epn/tmc* BGC is closely related to other *Streptomyces* BGCs linked to EPN and TMC specialized metabolites, as expected.

It is worth noticing that from all the genomes and BGC sequences evaluated (see “Materials and Methods” section for details) only 16 share sequence similarity and minimal BGC characteristics related to BRA-346 *epn/tmc* BGC. Even within 50 *Streptomyces* spp. closely related to BRA-346 (vide phylogenetic analysis of *Streptomyces* sp. BRA-346; Supplementary Figure S15) only two related BGCs could be found. Most of the organisms containing BGCs related to BRA-346 *epn/tmc* are of the phylum Actinobacteria, covering two orders: Streptomycetales and Pseudonocardiales. Interesting, *Coralloccocus* sp., and *Chondromyces* sp., both Proteobacteria in the order Myxococcales, and *Aquimarina* sp., a Bacteroidetes in the order Flavobacteriales, were also pointed as presenting a BGC related to the BRA-346 *epn/tmc* (Figure 6). This indicates the occurrence of putative epoxyketone peptide BGCs also in Gram negative bacteria. Together, these results indicated that this BGC is rare in nature, not frequent observed in closely related *Streptomyces* strains, and not very widespread across Bacteria, being so far restricted to three Phyla (Actinobacteria, Bacteriodetes, and Proteobacteria). The scarce presence of *epn/tmc* related BGCs in the BRA-346 species tree (Supplementary Figure S15), in addition to the finding that this BGC was found in unrelated Actinobacteria and Proteobacteria members (Figure 6), point to horizontal gene transfer of this BGC, which is a common feature of Actinobacteria BGCs linked to specialized metabolites (Ziemert et al., 2014).

Regarding the environment from which these bacteria or DNA samples were isolated, *Streptomyces* BRA-346 (marine endemic tunicate *Euherdmania* sp., Brazil), *Streptomyces luteovercillatus* CGMCC 15060 (ocean sediment, China) and *Aquimarina* sp. AU119 (Marine sponge *Tedania* sp., Australia) were isolated from the marine environment, the others being obtained from soil (most organisms, including eponemycin, TMC-86A, epoxomicin, clarepoxcin, and landepoxcin producers), river sediment (one organism), and decayed wood (one organism) samples.

Biosynthetically, the NRPS responsible for the biosynthesis of the peptide core of epoxyketone peptides present up to four condensation domains (C-domains; Supplementary Figure S15). Clades with BGCs sharing C-domain pattern similarity are indicated, as the NRPs encoding the tetrapeptides epoxomicin and clarepoxcin. One clade harbours BGCs with shorter NRPSs, with two C-domains, as Epn/TmcG and the NRPS from BRA-346 *epn/tmc* BGC, which are responsible for the production of the dipeptides eponemycin and TMC-86A. From these analyses it is possible to suggest that this BGC could suffer module duplication or loss in its evolutionary history.

The Epn/TmcG multi-modular enzymes, which add leucine and serine amino acids to a fatty acid precursor in EPN and TMC biosynthesis (Liu et al., 2015; Zabala et al., 2016), share higher sequence identity between *Streptomyces* BRA-346 Epn/TmcG and *S. hygroscopicus* EpnG (91% ID) than between BRA-346 Epn/TmcG and *S. chromofuscus* TmcG (76% ID). In BRA-346, Epn/TmcG might promiscuously accept both longer (EPN) and shorter (TMC) fatty acid precursors, therefore being able to synthesize both specialized metabolites. This potentially indicates an evolutionary path for this enzyme on the flexibility for TMC or EPN biosynthesis, a feature that has been involved in the “evolutionary dynamics of specialized metabolites biosynthesis” (Chevrette et al., 2020).

A common characteristic of the retrieved BGCs is the presence of a ACAD enzyme (ORF coloured yellow in Figure 6). The ACAD is related to Epn/TmcF in BRA-346 *epn-tmc* BGC and is linked to the biosynthesis of the epoxyketone pharmacophore in epoxyketone peptide proteasome inhibitors as EPN (Zettler et al., 2016) and TMC (Zabala et al., 2016). Except for the ACAD from *Chondromyces apiculatus* (NZ_ASRX01000038), the other ACAD enzymes showed at least 65 sequence similarity (Supplementary Table S8) reflecting a similar biosynthetic role.

From the 17 BGCs analyzed, nine also present an ORF encoding for a beta-subunit of the proteasome (related to Epn/TmcC; Figure 6B in cyan). This suggests the presence of a self-resistant gene in these BGCs and, possibly, the biological target for the resulting biosynthetic product (D’Costa et al., 2011; Kale et al., 2011; Ziemert et al., 2016; Alanjary et al., 2017). Indeed, six of the retrieved BGCs have a specialized metabolite assigned, which is proteasome inhibitors: eponemycin, TMC-86A, landepoxcin A, epoxomicin, and clarepoxcin C (Figure 6C). From these, the epoxomicin BGC was the only one that did not show a proteasome beta-subunit within the BGC.

Interesting, orphan BGCs from three other *Streptomyces* species (*Streptomyces* sp. NBS 14/10, *S. bingchenggensis* BCW-1, and *S. sparsogenes* ATCC 25498) and the BGC from *Actinocrispum wychmicini* DSM 45934 also carry a beta-subunit of the proteasome, showing high level of synteny to the *epn/tmc* BGCs. The first three orphan BGCs are further clustered with the epoxomicin and clarepoxcin C BGCs in the phylogenetic tree (Figure 6). This indicates that these Actinobacteria BGCs also produce epoxyketone peptides, potentially acting as proteasome inhibitors, thus deserving future research.

Regarding BGC regulation, all BGCs retrieved show regulatory genes flanking the BGC, which belong to a diverse set of transcriptional regulators (Figure 6). The three *epn/tmc* BGCs show regulatory genes upstream the BGC, belonging to the LuxR-type of transcription factors with HTH DNA-binding domains (Epn/TmcA). These DNA binding domains are conserved among the three Epn/TmcA sequences analysed (Supplementary Figure S17). BRA-346 Epn/TmcA is the longer sequence with 190 amino acids, displaying an additional PAS-fold regulatory domain at the N-terminal region of the coded protein. A deep inspection of the *epn* BGCs also reveals a PAS domain 275–20 bases upstream the *epnA* ORF. The same analyses could not be performed to the *tmc* BGC as its sequence is truncated just upstream the *tmcA* ORF in the public databases consulted. It can be suggested, at least for the BRA-346 *epn/tmc* BGC, that the PAS domain is an additional sensor for tight regulation of this BGC transcription. The BRA-346 Epn/TmcA PAS domain is predicted to bind at least one ligand, being a heme or flavin ligands (Supplementary Figure S18), which are related to light or redox regulation, respectively. PAS domains can also bind quorum sensing molecules (Pappas et al., 2004), a property that cannot be ruled out to BRA-346 Epn/TmcA at this point.

Although little is known about LuxR regulation in Gram-positive bacteria (Santos et al., 2012; Polkade et al., 2016; Rajput and Kumar, 2017), there are precedents in the literature that salinity increases the production of LuxR transcription factor activator molecule, acyl homoserine lactone (AHL), which in turn is produced by LuxI in Gram-negative bacteria (Bjelland et al., 2012; Sivakumar et al., 2019). Phylogenetic analysis of Gram-positive bacteria, however, point that the LuxI/LuxR system is absent in this group, although an analogous signalling molecule, gamma-butyrolactone (GBL), is found in Gram-positive bacteria (Santos et al., 2012; Rajput and Kumar, 2017). Indeed, we could not find a *luxI* related gene in BRA-346 genome, but we found two butyrolactone BGCs in BRA-346 genome (region 35.1, candidate BGC = 11 kb; and region 2.1, candidate BGC = 11 kb; Supplementary Figure S10). As mentioned above, these GBL BGCs are most certainly related to lipopeptides and macrolactam BGC activation in BRA-346, which in turn shuts-off the production of epoxyketone peptides by this bacterium. It is not clear however if these GBLs, or associated regulatory proteins, have a direct role on the downregulation of the *epn/tmc* BGC or if its downregulation is simply a consequence of biosynthetic precursor availability, once it competes with the GBL-regulated BRA-346 BGCs for amino and fatty acids.

In addition to LuxR transcriptional regulation, the *epn/tmcA* gene displays three TTA codons, further indicating regulation of this BGC at the translational level. The TTA codon is a rare codon in GC rich genomes, as those of Actinobacteria, which is linked to secondary metabolism regulation (Takano et al., 2003; Sen et al., 2012). Apart from *Aquamarina* sp. AU119 (33%), the other BGCs analysed here show high GC content (>67%) and TTA regulatory codons were frequently found within the epoxyketone peptide related BGCs, some of them also within the ORF encoding a transcription factor. Considering that Epn/TmcA is a positive regulator of the *epn/tmc* BGC, the TTA codon on its regulator is also a point of competition during translation. In summary, the analyses of the 16 BGCs related to BRA *epn/tmc* BGC suggest this BGC is tightly regulated, at both the transcription and translational levels, what can further explain the low reproducibility of wild type BRA-346 cultures in producing epoxyketone peptides.

## Discussion

*Streptomyces* sp. BRA-346 is a bacterium of pharmacological importance, as it has shown the capacity to reduce glioma cell viability (Furtado et al., 2021). In the latter report, BRA-346 crude extract and derived fractions were also described as containing epoxyketone peptides and to inhibit the 20S proteasome enzymatic activity. The analysis of the cellular pathways that lead to BRA-346 cell-killing effects in glioma cells indicates a molecular fingerprint related to proteasome inhibition. However, BRA-346 metabolic pools were more effective in killing glioma cells than the isolated DHE (**1**) alone (Furtado et al., 2021). This reflected that the pool of epoxyketone peptides produced by this bacteria, or other classes of secondary metabolites it could produce, have improved proteasome inhibition or synergistic effect on the latter biological phenomena.

Here, we show that BRA-346 has the genetic capacity to produce epoxyketone peptides, lipopeptides, macrolactams, carotenoids, diketopiperazines, macrolides, and other classes of secondary metabolites (Supplementary Table S6; Figure 2A). It is stressed from the data presented here that specialized metabolites production by BRA-346 is tightly regulated (Figure 2A), involves quorum sensing systems (Table 1) and can be controlled by specific growth conditions (Figure 2A; Supplementary Figure S9). In addition, the expression of BGCs under the control of TetR-family of transcription factors—as lipopeptides, macrolactams, diketopiperazines, and carotenoids—is correlated with a shift in BRA-346 metabolism directed to downregulation on the production of the target epoxyketone peptides. The latter, are encoded by a single biosynthetic gene cluster in BRA-346 (Figure 4) and this BGC can be expressed in the host organism *S. coelicolor* M1146, rendering compounds **1**–**4** and additional analogues (Figure 5). As *S. coelicolor* M1146 is an “antibiotic null” strain (Gomez-Escribano and Bibb, 2011) and the cloned *epn/tmc* BGC is under the control of a strong promoter, this strategy appears to overcome the negative regulation the other antibiotic BGCs of wild-type *Streptomyces* sp. BRA-346 exerts over the *epn/tmc* BGC. This might be due to increased biosynthetic precursors availability (e.g., amino acids and fatty acids), or by reducing quorum sensing and transcription regulation crosstalk.

In our attempts to use chemical elicitors to awake silent BGCs in BRA-346 ampicillin was the chemical elicitor tested that clearly modulated BRA-346 secondary metabolism in the direction of epoxyketone peptide production (from now on assigned as BRA-346 metabolites of group 1, G1). Ampicillin, a beta-lactam antibiotic produced by fungi, has been shown to awake cryptic BGC in bacteria (Okada and Seyedsayamdost, 2017; de Felício et al., 2021), suggesting a chemo-ecological role of ampicillin in eliciting antibiotic production by bacteria. This was also the case for BRA-346 in producing epoxyketone peptides (Figure 3). However, it is still unclear how beta-lactam antibiotics work as chemical elicitors at the molecular level and, in the case of BRA-346, it cannot yet be directly linked to broad epigenetic modulation or specific BGC regulation. Oppositely, the chemical elicitor procaine, upregulated carotenoids, macrolides, and some poorly annotated diketopiperazines/alkaloids (Supplementary Figure S9) in BRA-346 cultures using the A1 medium. This group of specialized metabolites, which can be further modulated by chemical elicitors in A1 medium, is assigned group 2 (G2). On the other hand, macrolactams, lipopeptides, and diketopiperazines related to bicyclomycin would constitute a third group (assigned as group 3, G3), apparently modulated by culture media containing low molecular weight carbohydrates, as TSBY and ISP2 media. The latter metabolites are linked to BRA-346 BGCs that present specific GBL regulatory systems, and TetR repressors (Table 1), linking the activation of GBL-controlled BGCs to nutrient usage, as previously reported (Du et al., 2011).

Interesting, the BGCs competing for epoxyketone production by wild type BRA-346—which are activated in TSBY and ISP2 media (G3)—are under the control of transcription factors predicted as TetR repressors; whereas competing BGCs activated in the A1 medium (G2, C5 or procaine) are under the control of TetR activators (Table 1). The *epn/tmc* BGC itself is also controlled by a transcription factor activator (LuxR, Epn/TmcA), also within the TetR superfamily.

Based on the data given, we hypothesize that the A1 medium is a condition in which quorum sensing molecules, as GBLs, are not being overproduced. The specialized metabolites in G3 are mostly repressed, with their TetR repressors blocking transcription. On the other hand, upon activation of GBL production, in ISP2 and TSBY media for example, these quorum sensing molecules can bind to the TetR repressors, releasing transcription of group 3 BGCs and, additionally, activating their SARP systems. As a result, G3-related BGCs are overexpressed and compete with G1 (epoxyketone peptides) for biosynthetic precursors.

The BGCs involved in the biosynthesis of G1 and G2, on the other hand, are mostly controlled by transcription activators. In these cases, a basal production is observed, with the preference for G1 or G2 biosynthesis controlled by the extent of activation these BGCs might have procaine upregulated G2 and ampicillin G1. It is not yet clear the exact molecular mechanisms underlying the observed regulation and future work would be necessary to decipher this thread at the molecular level. We could point, however, that the *epn/tmc* LuxR transcription factor with PAS domain (Epn/TmcA) would be controlled by broader biological pathways (e.g.: redox balance and iron/heme availability or light), as redox cofactors and heme are predicted as the ligands of the Epn/TmcA regulator.

By cloning the BRA-346 *epn/tmc* BGC into *S. coelicolor* M1146, we obtained epoxyketone peptides **1**, **3**, and **4** and several congeners, including eponemycin (**2**, Figure 5). Importantly, compounds **2** and **4** could be observed in high amounts, being clearly detected as high intensity LC–MS peaks in the crude extract of *S. coelicolor* M1146-*epn/tmc* (Figure 5C). This shows the efficiency and viability of BRA-346 *epn/tmc* BGC heterologous expression allowing the production of TMC-86A, eponemycin, and epoxyketone peptide congeners under laboratory conditions for future studies. The removal of competing BGCs in the *S. coelicolor* M1146 host organism might be one of the causes for the observed overexpression of epoxyketone peptide by the heterologous organism. The role of the full-length Epn/TmcA transcription factor present in this clone remains to be elucidated and explored in future efforts. In addition to the *epn/tmc* BGC overexpression on a heterologous host, this data also highlights that a single BGC of BRA-346 can produce at least two biosynthetic final products, being compounds **2** and **4**, showing the plasticity of the BRA-346 *epn/tmc* BGC to produce epoxyketone peptides.

Curiously, the epoxyketone peptides TMC-86A (**4**, *m/z* 343.19) and eponemycin (**2**, *m/z* 399.25) are produced by the heterologous organism containing the BRA-346 *epn/tmc* biosynthetic gene cluster, whereas the dihydro-intermediates dihydroeponemycin (**1**, *m/z* 401.26) and dihydro TMC-86A (**3**, *m/z* 345.20) are the preferred products of the wild type organism BRA-346 (Figure 5). It is reported that the oxireductase TmcK is the enzyme responsible for Leu oxidation of **1**, rendering **2** (Zabala et al., 2016). It is not clear; however, the mechanisms by which the final biosynthetic step of **2** and **4** is being regulated by BRA-346, since the cultures of the wild type organism favoured the detection of the n-1 dihydro-intermediates. This could be through gene expression of BRA-346 *epn/tmcK*, negative feedback inhibition of Epn/TmcK enzyme by the end product, or rapid degradation of **2** and **4** in BRA-346 cultures. Curiously, in our phylogenetic analyses we observed that *tmcK* also shows a TTA codon within the ORF, pointing to an additional regulatory mechanism at the translational level (in a biosynthetic enzyme) in the evolutionary history of this BGC.

## Conclusion

This work sheds light on the broad biosynthetic potential of *Streptomyces* sp. BRA-346 and on the mechanisms underlying the regulation of its secondary metabolism. It further advances the knowledge on the phylogenetic relationships of this species and, especially, on the epoxyketone peptide *epn/tmc* BGC. Genome sequencing and mining of BRA-346 revealed a broad biosynthetic capacity, including but not limited to three main groups of specialized metabolites that are potentially regulated together: group 1: epoxyketone peptides, group 2: lipopeptides, macrolactams and diketopiperazine related to bicyclomycin; group 3: macrolides, carotenoids, and some diketopiperazine/alkaloids. The production of the different classes of secondary metabolites by BRA-346 was modulated under laboratory conditions, being influenced by chemical elicitors in A1 medium cultures (ampicillin and procaine—e.g., group 1 vs. group 2) and different culture media (salt concentration and carbohydrate sources—e.g., group 1 vs. group 3). By cloning the *epn/tmc* BGC of BRA-346 into a host organism, originally uncapable of producing epoxyketone peptides, free of competing BGCs for biosynthetic precursors, reduced quorum sensing crosstalk and using a strong promoter in the *epn/tmc* cloning cassette, it was possible to produce the pharmacologically important eponemycin and TMC-86A in high yields. Heterologous expression of the single epoxyketone peptide BGC of BRA-346, *epn/tmc* BGC, shows the plasticity of this BGC in producing both eponemycin and TMC-86A, and additional congeners. Importantly, the biosynthetic final products of the *epn/tmc* BGC were preferentially detected in the heterologous organism, further stressing that these BRA-346 BGC biosynthetic products are under tight regulatory control in the wild type organism. Phylogenetic analyses of the *epn/tmc* BGC give further insights on the preference for **2** or **4** biosynthesis and points some of the molecular mechanisms underlying the tight regulation of the *epn/tmc* BGC. A LuxR transcription factor containing a PAS-regulatory domain, aligned with the presence of TTA codons, are proposed as the main mechanisms of regulation at the transcriptional and translational levels. Heterologous expression of BRA-346 *epn/tmc* BGC with truncated versions of the Epn/TmcA LuxR/PAS transcription factor, as well eventual artificial control of its full-length LuxR transcription factor at the protein level, are alternatives for further improving biotechnological production of epoxyketone peptides encoded by the *epn/tmc* BGC.

## Data Availability Statement

The datasets presented in this study can be found in online repositories. The names of the repository/repositories and accession number(s) can be found at: https://massive.ucsd.edu/, MSV000088148; MSV000088149; MSV000088150; and MSV000088812.

## Author Contributions

DB and BD: conceptualization, writing—original draft preparation, and writing—review and editing. BD, RF, CF, LC, RS, GF, LC, and HN: methodology. DB and RF: validation. RF, LG, and BB: formal analysis. RF, BD, HN, and DB: investigation. DB, LVC-L, and LC: resources. RF and BD: data curation. BD, DB, RF, and HN: visualization. DB and LVC-L: supervision and funding acquisition. DB: project administration. All authors contributed to the article and approved the submitted version.

## Funding

This research was funded by the Serrapilheira Institute, grant number Serra-1709-19681 (to DB) and the São Paulo Research Foundation (FAPESP), grants number 2019/27306-9 (to DB) and 2015/17177-6 (to LVC-L). Fellowships were supported by the National Council for Scientific and Technological Development (CNPq) to BD (870001/2017-5), to LC (140146/2020-2), and to LVC-L (306913/2017-8), the Serrapilheira Institute (to LG, CF, RS, and BD), and FAPESP to LC (2017/18235-5 and 2020/08987-2).

## Conflict of Interest

The authors declare that the research was conducted in the absence of any commercial or financial relationships that could be construed as a potential conflict of interest.

## Publisher’s Note

All claims expressed in this article are solely those of the authors and do not necessarily represent those of their affiliated organizations, or those of the publisher, the editors and the reviewers. Any product that may be evaluated in this article, or claim that may be made by its manufacturer, is not guaranteed or endorsed by the publisher.

## Footnotes

^1^ https://www.bioinformatics.babraham.ac.uk/projects/fastqc/

^2^ https://bitbucket.org/cnpemlqpn/np3_ms_workflow/src/master/

^3^ https://github.com/cytoscape/py2cytoscape
