# Supplementary material for: Production of Epoxyketone Peptide-Based Proteasome Inhibitors by Streptomyces sp. BRA-346: Regulation and Biosynthesis
Source: Front Microbiol. 2022 Mar 24;13:786008. doi: 10.3389/fmicb.2022.786008 (PMC8988807; doi:10.3389/fmicb.2022.786008)
Supplement: Supplementary file 1 [file Data_Sheet_1.docx]

Supplementary Material

**Production of epoxyketone peptides proteasome inhibitors by *Streptomyces* sp. BRA-346: regulation and biosynthesis**

Bruna Domingues Vieira^1,2^, Henrique Niero^1,2^, Rafael de Felício^1^, Luiz Fernando Giolo Alves^1^, Cristina Freitas Bazzano^1,3^, Renata Sigrist^1^, Luciana Costa Furtado­^4^, Gabriela Felix Persinoti^5^, Leticia Veras Costa-Lotufo^4^, Daniela B. B. Trivella^1*^

^1^ Brazilian Biosciences National Laboratory (LNBio), Brazilian Center for Research in Energy and Materials (CNPEM), Campinas, Brazil.

^2^ Faculty of Pharmaceutical Sciences (FCF), University of Campinas (UNICAMP), Campinas, Brazil.

^3^ Institute of Computing (IC), University of Campinas (UNICAMP), Campinas, Brazil.

^4^ Department of Pharmacology, Institute of Biomedical Sciences, University of São Paulo, São Paulo, Brazil.

^5^ Brazilian Biorenewables National Laboratory (LNBR), Brazilian Center for Research in Energy and Materials (CNPEM), Campinas, Brazil.

# Supplementary Methods

## TAR cloning and intergeneric conjugation for heterologous expression of *epn/tmc* BGC

Genomic and plasmid DNA were isolated using Quick-DNA™ Miniprep Plus Kit (Zymo Research), and QIAprep Spin Miniprep Kit (Qiagen). Primer pairs were designed to amplify six fragments of 3800 to 4800 bp with 150 bp homology to each other covering the *epn/tmc* BGC (26.5 kb). Initial and final fragments were amplified with overlapping arms (45 bp) to the vector pCAP03_p21 (Studier, 2005; Zhang et al., 2019) – Figure S1. Oligonucleotides used in this study are listed in Table S3. Fragments were amplified by PCR from genomic DNA using the Phusion High-Fidelity DNA polymerase with GC Buffer (Thermo Fisher Scientific), following the standard protocol recommended by the manufacturer. Betaine 1 M and DMSO 2.4% were used as additives of the PCR. Fragments were concentrated and purified from 0.5% agarose gels, using the Wizard SV Gel and PCR Clean-Up systems (Promega). The fragments were confirmed by restriction analysis (NdeI, EcoRV, EcoRI, and BamHI) – Figure S2. The plasmid pCAP03_p21 was linearized by EcoRI and NdeI. All restriction enzymes were purchased from Promega. The Geneious software package (Biomatters Ltd.) was used for molecular biology and sequencing analysis.

The highly transformable *Saccharomyces cerevisiae* VL6-48N (Kouprina and Larionov, 2006) (Table S1) was used for transformation to clone *epn-tmc* BGC. VL6-48N were cultivated in YPD medium (Table S2) supplemented with 100 mg/L adenine at 30 °C and 220 rpm for 24 hours. Spheroplasts were prepared using zymolyase solution (Zymolyase-20T 10 mg/mL, glycerol 50%, Tris 50 mM pH 7.5) as described by Zhang et al., 2019. Six purified DNA fragments of *epn/tmc* BGC (100 ng each) and pCAP03_p21 linearized vector (120 ng) were co-transformed into spheroplasts. After transformation, the cells were treated with an osmotic solution (1 M sorbitol) for cell wall recovery. Transformants were selected on tryptophan deficient selective plates (Bottom/Top Selective Agar – Table S2). After five days incubated at 30 °C, colonies were transferred to new selective medium plates. Colonies were screened by colony PCR using TAR_3F/2F, TAR_4F/3R, and TAR_5F/4R primer pairs to amplify the homology region between fragments 2/3 (142 bp), 3/4 (170 bp), and 4/5 (137 bp).

Positive colonies were cultivated in selective medium at 30 °C and 220 rpm for three days and DNA extraction was performed using phenol-chloroform extraction. pCAP03_p21-*epn/tmc* plasmids were introduced into *E. coli* TOP10 competent cells by electroporation protocol (Nováková et al., 2014). TOP10 colonies were confirmed by colony PCR and plasmids were isolated from positive transformants. pCAP03_p21-*epn/tmc* plasmids were transformed into *E. coli* DH5α to evaluate stability of plasmid constructed and plasmid storage. Plasmids were isolated to confirm vector construction by restriction analysis (HindIII, HindIII + EcoRI, EcoRI, XhoI + XbaI, and SpeI + KpnI) - Figure S3. *E. coli* strains were grown on LB medium at 37 °C and 200 rpm.

Triparental conjugation was performed between *E. coli* DH5α-pCAP03_p21-*epn/tmc* (donor, Kan^R^)*,* TOP10-pR9406 (helper, Carb^R^), and non-methylating ET12567 (aceptor, Cam^R^) on LB plates without antibiotics at 37 °C for 24 h. Exconjugants were isolated using appropriated antibiotics. Some colonies were chosen to confirm the presence of BGC by colony PCR. pCAP03_p21-*epn/tmc* plasmid was transferred into *Streptomyces coelicolor* M1146 (Gomez-Escribano and Bibb, 2011) by intergeneric conjugation carried out on solid SFM medium at 30 °C. Next day plates were fully covered with 1 mL appropriate antibiotic mixture to select exconjugants then incubated for six days. Exconjugants were isolated on a new SFM plate containing kanamycin and nalidixic acid (Figure S4). When appropriate, cultures were supplemented with the antibiotics for selection (50 μg/mL kanamycin, 25 μg/mL chloramphenicol, 100 μg/mL carbenicillin, 25 µg/µL nalidixic acid).

**Phylogenetic analysis of the BRA-346 *epn*/*tmc*, eponemycin, TMC-86A and related Biosynthetic Gene Clusters.**

MAFFT v11 alignment (Geneious Prime v.2021.2.2). Algorithm: Auto. Scoring Matrix: 200PAM / k=2. Gap Open Penalty: 1.53. OffSet value: 0.123.

MEGA v11 Maximum likelihood trees. Test of phylogeny: Bootstrap with 500 replications. Model of evolution: Generalized Time-Reversible model. Gaps/Missing Data Treatment: Use all sites. Initial Tree for ML: Make initial tree automatically, NJ/BioNJ. Branch swap filter: None. Rates among sites: Uniform rates. ML Heuristic method: Nearest-Neighbor-Interchange.

jModelTest2 model of evolution. Number of substitution schemes = 11. Including models with equal/unequal base frequencies (+F). Including models with/without a proportion of invariable sites (+I). Including models with/without rate variation among sites (+G) (nCat = 4). Optimized free parameters (K) = Substitution parameters + 17 branch lengths + topology. Base tree for likelihood calculations = Maximum LikelihoodTree topology search operation = NNI. Bayesian Information Criterion.

MultiGeneBlast. Number of Blast hits per gene to be mapped: 250. Weight of synteny conservation in hit sorting: 0.5. Minimal sequence coverage of BLAST hits: 25. Minimal % identity of BLAST hits: 30. Maximum distance between genes in licus (kb): 20.

# Supplementary Figures

Figure S1. Schematic view of fragments and primers designed to amplify the *epn/tmc* BGC from BRA-346 (26.4 kb).

**Figure S2.** **Restriction analysis of DNA fragments of *epn/tmc* BGC from *Streptomyces* sp. BRA-346.** Samples were digested with selected restriction enzymes for three hours at 37 °C. 0.5% agarose gel electrophoresis stained with ethidium bromide. 1 Kb Plus (Invitrogen) was used as DNA Ladder.

**Figure S3.** **Restriction analysis of plasmid pCAP03-*epn/tmc* (colonies A, B, C).** Samples were digested with selected restriction enzymes for three hours at 37 °C. 0.5% agarose gel electrophoresis stained with ethidium bromide. 1 Kb Plus (Invitrogen) was used as DNA Ladder.

**Figure S4. pCAP03-*epn/tmc* integration into *S. coelicolor* M1146 chromosome. (A)** Isolation of *S. coelicolor* M1146-*epn/tmc* exconjugants in SFM agar supplemented with kanamycin and nalidixic acid. **(B)** Confirmation of integration by DNA amplification of overlapping between fragments 2/3 (*primers* 3F/2R, 142 bp), 3/4 (*primers* 4F/3R, 170 bp), and 4/5 (*primers* 5F/4R, 137 bp). 1% agarose gel electrophoresis stained with ethidium bromide. 1 Kb Plus (Invitrogen) was used as DNA Ladder.

Figure S5. MS^2^ spectra of the ion *m/z* 401.26 at *t*_R_ 6.6 and 7.4 min obtained from BRA-346 C3b extract.

**Figure S6.** EIC *m/z* 399.25 ± 0.01 obtained from BRA-346 crude extract. The EPN retention time is shown, highlighting this ion is not observed in the BRA-346 cultures prepared in this work.

**Figure S7.** MS^1^ and MS^2^ of chromatographic peaks detected of *m/z* 399.25. **(A)** MS^1^ scan signals of EIC *m/z* 399.24 *t_R_* 8.5 min from C3_A1a. **(B)** MS^2^ spectra of *m/z* 399.2489 *t_R_* 7.3 min from C3_A1b. **(C)** MS^1^ scan signals of EIC *m/z* 399.24 *t_R_* 6.7 min and *t_R_* 7.3 min from C3_A1c. **(D)** MS^1^ scan signals of EIC *m/z* 399.24 *t_R_* 9.7 min.

**Figure S8.** EIC *m/z* 343.19 ± 0.01 obtained from BRA-346 crude extract. Peaks are detected at retention time of TMC-86A, *t*_R_ 4.5 and 4.7 min, however the fragmentation spectra were not obtained due to low intensity of the MS^1^ peak.

**Figure S9.** **BRA-346 secondary metabolism is modulated by chemical elicitors in the A1 media.** Figure shows a simplified SSMN of BRA-346 cultures using the A1 medium and different chemical elicitors (no elicitor, as a control, (i); ampicillin 100 µg/mL (ii); sodium butyrate 50 µM (iii); procaine 100 µM (iv)). Only MS/MS clusters assigned to [M+H]^+^ spectra and annotated as secondary metabolites are shown. Only nodes with at least two connections (clusters) are shown for clarity. Data for the C5 experiment, crude extracts.

**Figure S10.** Butyrolactone BGCs found in BRA-346 genome.

**Figure S11.** Characterization and identification of lipopeptides stendomycin through NP^3^_MS_workflow and NP^3^ mirror comparison spectra tool. **(A)** Base peak chromatogram (m/z 200 to 2000 Da) of organic extract from BRA346 strain growth in ISP2 medium, highlighting complex overlapped peaks between retention time 11.7 and 14.0 min. **(B)** Respective MS^1^ scan signals of chromatographic peak mentioned (A), showing *m/z*s featured double charged around *m/z* 800-850 Da. **(C)** and **(D)** frames – NP^3^ mirror comparison spectra tool in which top spectra (black lines) are MS^2^ scans from BRA-346-ISP2_extract UPLC-MS/MS analysis, and bottom spectra (red lines) are spectra from Stendomycin-like compounds obtained from GNPS (https://gnps.ucsd.edu/ProteoSAFe/gnpslibrary.jsp?library=all#%7B%22main.Compound_Name_input%22%3A%22Stendomycin%22%7D, accessed in 10/09/2021). **(C)** MS^2^ Scan 4851 (*m/z* 801.0075) x GNPS CCMSLIB0000075353 Stendomycin (SHY-1600); **(D)** MS^2^ Scan 4851 (*m/z* 808.0142) x GNPS CCMSLIB0000075356 Stendomycin (SHY-1614); **(E)** MS^2^ spectra of other two lipopeptide derivatives: (scan 4851 (*m/z* 801.0075) - identified through NP^3^_MS_workflow and GNPS database; scan 5205 (*m/z* 808.0142) - identified through NP^3^_MS_workflow and GNPS database; scan 5412 (*m/z* 815.0235) - analogue compound; scan 5537 (*m/z* 822.0306) - analogue compound); **(F)** Stendomycins I-VI identified by Kersten and coworkers (2011) in a mass spectrometry-guided genome mining approach, from *Streptomyces hygroscopicus* ATCC 53653 (Kersten et al., 2011).

**Figure S12.** Stendomycin elucidation based on MS2 data. **(A)** Overview of the b- and y- fragmentation series for stendomycin congeners (Kersten et al., 2011; Villadsen et al., 2018). **(B)** Proposed structure (hidrolyzed form) for *m/z* 808.0142 based on previous work and Table S5.

**Figure S13.** **Spectra similarity molecular network (SSMN) of heterologous expression of the *epn/tmc* BGC of BRA-346.** MS/MS spectra detected in the crude extracts from wild type bacterium BRA-346 (pink), host organism M1146 (dark purple), and heterologous organism M1146-*epn/tmc* (purple) are shown.

**Figure S14.** MS^2^ spectra of the ions *m/z* 343 (*t*_R_ 4.7 min), *m/z* 345 (*t*_R_ 4.2 min), *m/z* 399 (*t*_R_ 7.9 min), and *m/z* 401 (*t*_R_ 7.4 min) obtained from M1146-*epn/tmc* extract.

**Figure S15. Phylogenetics analyses of *Streptomyces* sp. BRA-346.** Phylogenetic species tree was constructed with the Automated Multi-Locus Species Tree (autoMLST) webserver (Alanjary et al., 2019) using the *Streptomyces* sp. BRA-346 draft genome as the query sequence. The source genome (query sequence, QS) is highlighted in bold with the epoxyketone *epn/tmc* BGC represented in the right side of the tree. The BGCs of related *Streptomyces* species showing similar ACADs are also highlighted with their extracted BGCs in the right.

**Figure S16. Condensation domains in Epn/TmcG and related NRPSs.** Biosynthetically related clades are indicated: (A) eponemycin and TMC-86A, (B) epoxomicin and Clarepoxcin C, and (C) Landepoxcin A. Domains identified with the antiSMASH pipeline. C-domains and their respective NRPS are highlighted in the BGC using Geneious Prime (v 2021.2.2) software.

**Figure S17. Sequence alignment of LuxR transcription factors encoded by *epn/tmcA* ORF in the epoxyketone peptide BGCs of *S. chromofuscus* (TmcA), *S. hygroscopicus* (EpnA) and *Streptomyces* sp. BRA-346 (Epn/TmcA).**

**Figure S18. In silico modelling of the LuxR-type transcription factors investigated in the present work.** (**A**) Three dimensional models constructed to the LuxR-type transcription factors found in of *S. chromofuscus* (TmcA, in green), *S. hygroscopicus* (EpnA, in cyan) and BRA-346 (Epn/TmcA, in magenta). The ligand binding site in the PAS-domain of BRA-346 Epn/TmcA suggested by Phyre2 (Kelley et al., 2015), 3D ligand site (Wass et al., 2010) and COACH (Wu et al., 2018) servers are highlighted in white, blue and orange surfaces, respectively. The Phyre2 models used were 4YN8, 4HYE and 3CLO, for TmcA, EpnA and Epn/TmcA respectively. (**B**) Ligand predicted binding site for BRA-346 Epn/TmcA suggested by the 3D ligand site server with FAD, FMN or heme ligands docked. The ligands are shown in pale green sticks. Binding site residues highlighted in (A) are: Phyre2 pocket: 50-57, 64-68, 84-86, 102, 106-109 and 11-113; 3DLigandSite: 8, 10, 39, 42, 43, 47, 48, 50-52, 54, 66-68, 70-72, 85-86, 101-103; COACH: 8,10,38,39,40,42,43,52,55,56,59,70,72,81,84,86,98,99,100,102.

**Figure S19. Location of TTA codons in the retrieved BGCs.**

# Supplementary Tables

**Table S1.** Strains and plasmids used in this study.

| **Strain or plasmid** | **Relevant characteristics** | **Reference** |
| --- | --- | --- |
| ***Strains*** | | |
| *Streptomyces* sp. BRA-346 | Native producer of epoxyketone peptides | Furtado et al., 2021 |
| *S. cerevisiae* VL6-48N | Host strain for homologous recombination (MATα, his3∆200, trp1-∆1, ura3-∆1, lys2, ade2-101, met14, cir°) | Kouprina and Larinov, 2006 |
| *E. coli* DH5α | Plasmid transformation and storage (F– endA1 glnV44 thi-1 recA1 relA1 gyrA96 deoR nupG purB20 φ80dlacZΔM15 Δ(lacZYA-argF)U169, hsdR17(rK–mK+), λ–) |  |
| *E. coli* TOP10 | Plasmid transformation and storage (F- mcrA Δ(mrr-hsdRMS-mcrBC) φ80lacZΔM15 ΔlacX74 nupG recA1 araD139 Δ(ara-leu)7697 galE15 galK16 rpsL(Str^R^) endA1 λ–) |  |
| *E. coli* ET12567 | Donor strain for conjugation for triparental conjugation  (F-dam-13::Tn9 dcm-6 hsdM hsdR recF143 zjj202::Tn10 galK2 galT22 ara14 pacY1 xyl-5 leuB6 thi1) |  |
| *S. coelicolor* M1146 | Host strain for heterologous expression (Δact Δred Δcpk Δcda) | Gomez-Escribano and Bibb, 2011 |
| *S. coelicolor* M1146-*epn/tmc* | Heterologous host containing pCAP03_p21-*epn/tmc* | This study |
| ***Plasmids*** | | |
| pCAP03_p21 | Gene cluster capture vector (pCAP03-acc(3)IV derivative) | Tang et al., 2015 |
| pCAP03_p21-*epn/tmc* | pCAP03 derivative with *epn/tmc* gene cluster (Kan^R^) | This study |
| pR9406 (*E. coli* TOP10) | Helping plasmid for conjugation (derivative from pUB307, CarbR) | Piffaretti et al., 1988 |

**Table S2.** Composition of culture media used in this study.

| **Name** | **Medium component** |
| --- | --- |
| *A1* | 1% soluble starch, 0.4% yeast extract, 0.2% peptone, 2.9% reconstituted seawater |
| *A1 agar* | 1% soluble starch, 0.4% yeast extract, 0.2% peptone, 2.9% reconstituted seawater, 1.5% agar |
| *TSB* | 3% tryptic soy broth, 2% NaCl |
| *TSBY* | 3% tryptic soy broth, 10% sucrose, 0.5% yeast extract |
| *ISP2* | 0.4% yeast extract, 1% malt extract, 0.4% dextrose |
| *LB* | 1% peptone, 0.5% yeast extract, 1% NaCl |
| *LB agar* | 1% peptone, 0.5% yeast extract, 1% NaCl, 1.5% agar |
| *YPD* | 2% D-glucose, 1% yeast extract, 2% peptone |
| *YPD agar* | 2% D-glucose, 1% yeast extract, 2% peptone, 1.5% agar |
| *SFM agar* | 2% soy flour, 2% mannitol, 2% agar, 10 mM MgCl2 |
| *Top Selective agar* | 1 M sorbitol, 2% dextrose, 2% agar, 0.01% adenine, 0.1% 5-FOA*, 1X N-Bases** |
| *Bottom Selective agar* | 1 M sorbitol, 2% dextrose, 3% agar, 0.01% adenine, 0.1% 5-FOA*, 1X N-Bases** |

* 5-FOA (5-fluoroorotic acid)

** N-Bases (0.17% yeast Nitrogen base without amino acids and ammonium sulfate, 0.19% yeast synthetic drop-out medium supplements without tryptophan, 0.5% ammonium sulfate)

**Table S3.** Oligonucleotide primers used for the cloning and checking of the *epn/tmc* BGC.

| **Name** | **Primer sequence 5' → 3'** |
| --- | --- |
| TAR_1F­ | gtaggatgagcaatctagtcgagcaacggaggtacggac**gaattc**GTGAGGTCGGCCACCACCGCAAAGCTCCGC |
| TAR_1R | CTATCTCCCGATCGTCCGCGGATCCCAGTGCCCTCCGGCCCGCCTC |
| TAR_2F | TGTCACCAATGACGATCTGTGCGCC |
| TAR_2R | GCAGCGGCAGTACGTTGGAGAAGTA |
| TAR_3F | ATGATCCCGCTGTACGCCGCCTTCT |
| TAR_3R | TCTGCGACCGGAGTGGACAGATAGG |
| TAR_4F | AGCCATCTGTCCAGCGTGGGCTTC |
| TAR_4R | AGGGCATTGACCCTGACGCCCG |
| TAR_5F | TTGGCCGGGTGCCACCCGTTAC |
| TAR_5R | TGGTGGTCCCTGCTGCCCCAG |
| TAR_6F | TCGTCCTGCTGGCCGTCCTCAAGACCGGGGCCGC |
| TAR_6R | aggatgagtagcagcacgttccttatatgtagctttcga**catatg**GCGCGCCTGGTCAAGAGGCATATCTGTACT |

­­Lower case represents overlapping regions with flanking elements for capture vector (pCAP03).

Reconstituted restriction sites for EcoRI, and NdeI are shown in bold.

**Table S4. Node ([M+H]^+^) count per growth condition.** Exclusive nodes (nodes only detected in a particular condition) are also presented. Nodes relative to each culture media themselves were removed from the network.

|  | **Nodes** | |
| --- | --- | --- |
| Sample | Total | % |
| A1 a | 173 | 20.8 |
| A1 a exclusive | 52 | 6.3 |
| A1 b | 344 | 41.4 |
| A1 b exclusive | 81 | 9.8 |
| A1 c | 315 | 38.0 |
| A1 c exclusive | 49 | 5.9 |
| A1 a+b+c | 455 | 54.8 |
| A1 a+b+c exclusive | 309 | 37.2 |
| TSB | 143 | 17.2 |
| TSB exclusive | 67 | 8.1 |
| TSBY | 224 | 27.0 |
| TSBY exclusive | 132 | 15.9 |
| ISP2 | 274 | 33.0 |
| ISP2 exclusive | 142 | 17.1 |
| SSMN (M+H) | 830 | 100 |

**Table S5.** MS fragments of *m/z* 808.0142. Structure proposition (Figure S10) was made in accordance with Villadsen et al., 2018, considering the occurrence of hydrolyzation ESI source.

| **Precursor ion [*m/z*]: 808.0142 [M+2H^+^]** | | | | | | |
| --- | --- | --- | --- | --- | --- | --- |
| **Observed mass [M+H^+^]** | **Calculated mass [M+H^+^]** | **Difference [Da]** | **Error (ppm)** | **Species** | **Residue** | **Putative**  **amino acid** |
| 201.1351 | 201.1346 | 0.0005 | 2.49 | y1 |  |  |
| - | 314.2187 | - | - | y2 | 113.0869 | Ile, Leu |
| 401.2484 | 401.2507 | -0.0023 | -5.73 | y3 | 87.032 | Ser |
| 502.2974 | 502.2984 | -0.0010 | -1.99 | y4 | 101.0477 | Thr |
| 601.3684 | 601.3668 | 0.0016 | 2.66 | y5 | 99.0684 | Val |
| 700.4422 | 700.4352 | 0.0070 | 9.99 | y6 | 99.0684 | Val |
| 783.4625 | 783.4723 | -0.0098 | -12.51 | y7-H_2_O |  |  |
| - | 801.4829 | - | - | y7 | 101.0477 | Thr |
| 866.5187 | 866.5094 | 0.0093 | 10.73 | y8-H_2_O |  |  |
| - | 884.5200 | - | - | y8 | 83.0371 | Dhb, NMeDha, HseL |
| 937.5503 | 937.5465 | 0.0038 | 4.05 | y9-H_2_O |  |  |
| - | 955.5571 | - | - | y9 | 71.0371 |  |
| - | 1068.6412 | - | - | y10 | 113.0841 | Ile, Leu |
| - | 1167.7096 | - | - | y11 | 99.0684 | Val |
| - | 1224.731 | - | - | y12 | 57.0214 | Gly |
| - | 1339.7944 | - | - | y13 | 115.0634 | NMeThr |
| - | 1436.8471 | - | - | y14 | 97.0527 | Pro |
| 70.065 | 70.0651 | -0.0001 | -1.43 | b1-CO-COC_12_H_25_ |  |  |
| 98.0591 | 98.0600 | -0.0009 | -9.18 | b1-COC_12_H_25_ |  | Pro |
| 266.2478 | 266.2478 | 0.0000 | 0.00 | b1-CO |  |  |
| 294.2426 | 294.2428 | -0.0002 | -0.68 | b1 |  | Pro+COC_12_H_25_ |
| 365.2795 | 365.2799 | -0.0004 | -1.10 | b2-C_2_H_5_O |  |  |
| 409.3062 | 409.3061 | 0.0001 | 0.24 | b2 | 115.0633 | NMeThr |
| 448.3159 | 448.3170 | -0.0011 | -2.45 | b3-H_2_O |  |  |
| - | 466.3275 | - | - | b3 | 57.0214 | Gly |
| 565.3935 | 565.3960 | -0.0025 | -4.42 | b4 | 99.0685 | Val |
| 678.4669 | 678.4800 | -0.0131 | -19.31 | b5 | 113.0840 | Ile, Leu |
| 749.529 | 749.5171 | 0.0119 | 15.88 | b6 | 71.0371 |  |
| - | 832.5543 | - | - | b7 | 83.0372 | Dhb, NMeDha, HseL |
| - | 915.5914 | - | - | b8-H_2_O |  |  |
| - | 933.6019 | - | - | b8 | 101.0476 | Thr |
| - | 1032.6703 | - | - | b9 | 99.0684 | Val |
| - | 1131.7388 | - | - | b10 | 99.0685 | Val |
| - | 1232.7864 | - | - | b11 | 101.0476 | Thr |
| - | 1319.8185 | - | - | b12 | 87.0321 | Ser |
| - | 1432.9025 | - | - | b13 | 113.0840 | Ile, Leu |

**Table S6. BGC mined from the BRA-346 draft genome.** Genome mining was performed using antiSMASH v. 6.0.1.

| **Region** | **Type** | **Size** | **Truncated** | **Most similar known cluster** | | **Similarity** |
| --- | --- | --- | --- | --- | --- | --- |
|  |  |  |  | **Coumpound** | **Type** |  |
| 2.1 | T1PKS, NRPS, PKS-like, butyrolactone | 107,590 | 0 | actinomycin D/ heronamide B | NRP + Polyketide | 7% |
| 2.2 | hglE-KS, T1PKS | 50,752 | 0 | meridamycin | NRP + Polyketide | 5% |
| 2.3 | RiPP-like | 10,224 | 0 | - | - | - |
| 2.4 | NAPAA | 33,941 | 0 | phthoxazolin | NRP + Polyketide | 9% |
| 3.1 | terpene | 21,196 | 0 | 2-methylisoborneol | Terpene | 100% |
| 3.2 | Other, CDPS | 43,669 | 0 | bicyclomycin | tRNA-derived | 100% |
| 4.1 | siderophore | 14,135 | 0 | - | - | - |
| 5.1 | T2PKS | 72,524 | 0 | spore pigment | Polyketide | 83% |
| 5.2 | transAT-PKS | 62,183 | 0 | 9-methylstreptimidone | T1PKS | 74% |
| 5.3 | lasso peptide | 26,225 | 0 | chloromyxamide | NRP | 6% |
| 5.4 | terpene | 26,762 | 0 | hopene | Terpene | 69% |
| 5.5 | NRPS-like | 42,813 | 0 | echoside A - E | NRP | 17% |
| 6.1 | ectoine | 10,404 | 0 | ectoine | Other | 100% |
| 8.1 | RiPP-like | 11,304 | 0 | - | - | - |
| 9.1 | NRPS-like | 43,143 | 0 | - | - | - |
| 10.1 | RRE-containing | 22,384 | 0 | granaticin | T2PKS | 10% |
| 12.1 | NRPS-like | 42,819 | 0 | echoside A - E | NRP | 82% |
| 14.1 | siderophore | 12,369 | 0 | ficellomycin | NRP | 3% |
| 15.1 | redox-cofactor | 22,094 | 0 | lankacidin C | NRP + Polyketide | 20% |
| 16.1 | nucleoside | 20,911 | 0 | - | - | - |
| 17.1 | lasso peptide | 22,556 | 0 | SSV-2083 | RiPP:Lanthipeptide | 18% |
| 20.1 | hglE-KS | 46,110 | 0 | meilingmycin | Polyketide | 2% |
| 20.2 | furan | 21,043 | 0 | yatakemycin | NRP | 9% |
| 21.1 | NRPS | 50,970 | 0 | coelichelin | NRP | 90% |
| 21.2 | T1PKS | 46,410 | 0 | salinomycin | T1PKS | 6% |
| 24.1 | terpene | 22,222 | 0 | geosmin | Terpene | 100% |
| 29.1 | terpene | 21,085 | 0 | pristinol | Terpene | 100% |
| 34.1 | terpene | 24,727 | 0 | isorenieratene | Terpene | 71% |
| 35.1 | butyrolactone | 10,938 | 0 | - | - | - |
| 35.2 | NRPS | 76,965 | 0 | dechlorocuracomycin | NRP | 8% |
| 38.1 | NRPS-like | 43,827 | 0 | cyphomycin | Polyketide | 2% |
| 39.1 | lanthipeptide-class-i | 26,071 | 0 | - | - | - |
| 42.1 | NRPS, T1PKS, NRPS-like | 41,550 | 0 | eponemycin | NRP + T1PKS | 85% |
| 45.1 | terpene | 22,255 | 0 | geosmin | Terpene | 100% |
| 46.1 | siderophore | 11,787 | 0 | desferrioxamin B | Other | 100% |
| 68.1 | betalactone | 22,583 | 0 | A54145 | NRP | 5% |
| 1.1 | lanthipeptide-class-i | 21,557 | 1 | - | - | - |
| 2.5 | T1PKS | 29,098 | 1 | nigericin | T1PKS | 38% |
|  |  |  |  |  |  |  |
| 12.2 | T1PKS | 37,590 | 1 | JBIR-100 | Polyketide:type I | 61% |
| 26.1 | RiPP-like | 7,483 | 1 | - | - | - |
| 32.1 | NRPS | 35,163 | 1 | oxalomycin B | NRP + Polyketide | 9% |
| 33.1 | NRPS | 50,450 | 1 | oxalomycin B | NRP + Polyketide | 12% |
| 35.3 | T1PKS, NRPS | 49,751 | 1 | nannocystin a | NRP + Polyketide | 28% |
| 36.1 | NRPS | 43,901 | 1 | ochronotic pigment | Other | 75% |
| 36.2 | ladderane,arylpolyene | 34,736 | 1 | atratumycin | NRP | 34% |
| 37.1 | transAT-PKS, NRPS, T1PKS | 56,521 | 1 | phthoxazolin | NRP + Polyketide | 6% |
| 47.1 | PKS-like | 32,695 | 1 | vicenistatin | Polyketide | 35% |
| 54.1 | transAT-PKS-like | 30,448 | 1 | - | - | - |
| 61.1 | T1PKS | 29,034 | 1 | niphimycins C-E | Polyketide | 25% |
| 63.1 | T1PKS | 27,295 | 1 | heronamide A - F | NRP + Polyketide | 70% |
| 64.1 | T1PKS | 26,916 | 1 | hitachimycin | Polyketide | 27% |
| 70.1 | T1PKS | 19,954 | 1 | - | - | - |
| 72.1 | T1PKS | 17,916 | 1 | nigericin | T1PKS | 83% |
| 75.1 | T1PKS | 15,784 | 1 | candicidin | Polyketide | 23% |
| 81.1 | T1PKS | 29,795 | 1 | streptolydigin | NRP + T1PKS | 7% |
| 86.1 | T1PKS | 15,802 | 1 | - | - | - |
| 88.1 | T1PKS | 10,162 | 1 | cyphomycin | Polyketide | 12% |
| 90.1 | T1PKS | 10,082 | 1 | - | - | - |
| 91.1 | T1PKS | 9,589 | 1 | - | - | - |
| 93.1 | T1PKS | 9,533 | 1 | lydicamycin | NRP + T1PKS | 24% |
| 96.1 | T1PKS | 8,164 | 1 | - | - | - |
| 105.1 | T1PKS | 5,283 | 1 | - | - | - |
| 134.1 | NRPS | 2,756 | 1 | luminmide | NRP | 100% |
| 147.1 | T1PKS | 2,007 | 1 | - | - | - |
| 156.1 | NRPS | 1,803 | 1 | luminmide | NRP | 100% |
| 170.1 | T1PKS | 1,342 | 1 | - | - | - |

**Table S7.  Open reading frames identified in the *epn/tmc* biosynthetic gene cluster of BRA-346.** The related ORFs reported for the eponemycin and TMC-86A epoxyketone gene clusters are annotated for comparison, along with their sequence identities to the *epn*^1^ and *tmc*^2^ ORFs.

| **ORF ID** | **Number of amino acids** | **ORF annotated function*** | **%ID to related proteins in *epn* and *tmc* clusters **** | |
| --- | --- | --- | --- | --- |
| BRA7281 | 317 | - |  | |
| BRA7282 | 132 | Periplasmic binding proteins, sugar binding domain of LacI family |  | |
| BRA7283 | 525 | Glycosyl hydrolases family 43 |  | |
| BRA7284 | 556 | Alpha galactosidase A; Ricin-type beta-trefoil lectin domain-like |  | |
| BRA7285 | 861 | NlpC/P60 family |  | |
| BRA7286 | 178 | - |  | |
| BRA7287 | 61 | - |  | |
| BRA7288 | 181 | Methyltransferase domain |  | |
| BRA7289 | 105 | - | orf -1 (12%) |  |
| BRA7290 | 191 | Bacterial regulatory proteins, luxR family | EpnA (54%) | TmcA (32%) |
| BRA7291 | 260 | Thioesterase | EpnB (98%) | TmcB (72%) |
| BRA7292 | 286 | Proteasome subunit | EpnC (100%) | TmcC (92%) |
| BRA7293 | 341 | Ketosynthase III | EpnD (97%) | TmcD (89%) |
| BRA7294 | 77 | Phosphopantetheine attachment site | EpnE (93%) | TmcE (82%) |
| BRA7295 | 600 | Acyl-CoA dehydrogenase | EpnF (98%) | TmcF (81%) |
| BRA7296 | 2138 | Non-ribosomal Peptide Synthetase | EpnG (91%) | TmcG (76%) |
| BRA7297 | 2022 | Polyketide Synthase | EpnH (97%) | TmcH (80%) |
| BRA7298 | 432 | Cytochrome P450 | EpnI (99%) | TmcI (87%) |
| BRA7299 | 659 | Adenylation domain | EpnJ (99%) | TmcJ (90%) |
| BRA7300 | 407 | Cytochrome P450 | EpnK (94%) | TmcK (93%) |
| BRA7301 | 96 | Hypotetical protein | EpnL (96%) | TmcL (9%) |
| BRA7302 | 257 | Integrase core domain | orf +1 (20%) | orf +1 (11%) |
| BRA7303 | 228 | - |  | |
| BRA7304 | 115 | - |  | |
| BRA7305 | 354 | Stage II sporulation protein E (SpoIIE) |  | |

*Deduced functions of ORFs in the *epn/tmc* BGC

**Amino acid sequence homology from *blastp*

^1^(Schorn et al., 2014), ^2^(Zabala et al., 2016)

**Table S8. Amino acid sequence identity and similarities for the ACAD enzymes present within BGCs related to BRA-346 *epn/tmc* BGC.**

|  | BRA-346 | | EpnF | | TmcF | |
| --- | --- | --- | --- | --- | --- | --- |
|  | %Identity | %Similarity | %Identity | %Similarity | %Identity | %Similarity |
| BRA-346 | 100 | 100 | 99 | 99 | 89 | 95 |
| EpnF | 99 | 99 | 100 | 100 | 89 | 95 |
| TmcF | 89 | 95 | 89 | 95 | 100 | 100 |
| NZ_NCSM01000022 | 43 | 74 | 43 | 74 | 44 | 74 |
| NC_016582 | 43 | 73 | 43 | 73 | 44 | 73 |
| NZ_MAXF0100013 | 44 | 74 | 44 | 74 | 45 | 73 |
| epxF_BGC0000346 | 47 | 77 | 46 | 77 | 46 | 77 |
| BGC0001203 | 46 | 73 | 46 | 73 | 45 | 74 |
| NZ_CP034587 | 43 | 70 | 42 | 69 | 42 | 71 |
| NZ_JOIU01000026 | 42 | 69 | 42 | 69 | 42 | 71 |
| BGC0001202 | 44 | 71 | 44 | 71 | 42 | 70 |
| NZ_SLWS01000004 | 43 | 71 | 43 | 71 | 43 | 70 |
| NZ_RAWR0100016 | 43 | 72 | 43 | 71 | 43 | 72 |
| NZ_JODT01000033 | 44 | 73 | 44 | 73 | 44 | 74 |
| NZ_LWLC01000022 | 39 | 69 | 38 | 69 | 39 | 70 |
| NZ_OMPF01000035 | 32 | 65 | 32 | 64 | 32 | 64 |
| NZ_ASRX01000038 | 17 | 44 | 17 | 43 | 18 | 45 |

# References

Alanjary, M., Steinke, K., and Ziemert, N. (2019). AutoMLST: an automated web server for generating multi-locus species trees highlighting natural product potential. *Nucleic Acids Res.* 47, W276–W282. doi:10.1093/nar/gkz282.

Furtado, L. C., Bauermeister, A., de Felicio, R., Ortega, R., Pinto, F. das C. L., Machado-Neto, J. A., et al. (2021). Marine Streptomyces sp. Isolated From the Brazilian Endemic Tunicate Euherdmania sp. Produces Dihydroeponemycin and Analogs With Potent Antiglioma Activity. *Front. Mar. Sci.* 8. doi:10.3389/fmars.2021.644730.

Gomez-Escribano, J. P., and Bibb, M. J. (2011). Engineering Streptomyces coelicolor for heterologous expression of secondary metabolite gene clusters. *Microb. Biotechnol.* 4. doi:10.1111/j.1751-7915.2010.00219.x.

Kelley, L. A., Mezulis, S., Yates, C. M., Wass, M. N., and Sternberg, M. J. E. (2015). The Phyre2 web portal for protein modeling, prediction and analysis. *Nat. Protoc.* 10, 845–858. doi:10.1038/nprot.2015.053.

Kersten, R. D., Yang, Y. L., Xu, Y., Cimermancic, P., Nam, S. J., Fenical, W., et al. (2011). A mass spectrometry-guided genome mining approach for natural product peptidogenomics. *Nat. Chem. Biol.* 7. doi:10.1038/nchembio.684.

Kouprina, N., and Larionov, V. (2006). TAR cloning: Insights into gene function, long-range haplotypes and genome structure and evolution. *Nat. Rev. Genet.* 7. doi:10.1038/nrg1943.

Nováková, J., Izsáková, A., Grivalský, T., Ottmann, C., and Farkašovský, M. (2014). Improved method for high-efficiency electrotransformation of Escherichia coli with the large BAC plasmids. *Folia Microbiol. (Praha).* 59. doi:10.1007/s12223-013-0267-1.

Schorn, M., Zettler, J., Noel, J. P., Dorrestein, P. C., Moore, B. S., and Kaysser, L. (2014). Genetic basis for the biosynthesis of the pharmaceutically important class of epoxyketone proteasome inhibitors. *ACS Chem. Biol.* 9, 301–309. doi:10.1021/cb400699p.

Studier, F. W. (2005). Protein production by auto-induction in high-density shaking cultures. *Protein Expr. Purif.* 41, 207–234. doi:10.1016/j.pep.2005.01.016.

Villadsen, N. L., Hansen, B. K., Svenningsen, E. B., Jørgensen, K. H., Tørring, T., and Poulsen, T. B. (2018). Stendomycin and Pantomycin Are Identical Natural Products: Preparation of a Functionalized Bioactive Analogue. *J. Org. Chem.* 83, 7303–7308. doi:10.1021/acs.joc.8b00553.

Wass, M. N., Kelley, L. A., and Sternberg, M. J. E. (2010). 3DLigandSite: predicting ligand-binding sites using similar structures. *Nucleic Acids Res.* 38, W469–W473. doi:10.1093/nar/gkq406.

Wu, Q., Peng, Z., Zhang, Y., and Yang, J. (2018). COACH-D: improved protein–ligand binding sites prediction with refined ligand-binding poses through molecular docking. *Nucleic Acids Res.* 46, W438–W442. doi:10.1093/nar/gky439.

Zabala, D., Cartwright, J. W., Roberts, D. M., Law, B. J. C., Song, L., Samborskyy, M., et al. (2016). A Flavin-Dependent Decarboxylase-Dehydrogenase-Monooxygenase Assembles the Warhead of α,β-Epoxyketone Proteasome Inhibitors. *J. Am. Chem. Soc.* 138, 4342–4345. doi:10.1021/jacs.6b01619.

Zhang, J. J., Yamanaka, K., Tang, X., and Moore, B. S. (2019). “Direct cloning and heterologous expression of natural product biosynthetic gene clusters by transformation-associated recombination,” in *Methods in Enzymology* doi:10.1016/bs.mie.2019.02.026.
